# Excitation of Tamm plasmon polariton in ultrathin metals

**DOI:** 10.1126/sciadv.adz0106

**Published:** 2025-10-22

**Authors:** Jiangwei Zhang (张江伟), Pengsen Wang (王鹏森), Maobin Xie (谢茂彬), Qingquan Liu (刘清权), Anping Ge (葛安萍), Xueyu Guan (关学昱), Hengyi Cui (崔恒毅), Qixiang Jia (贾奇祥), Ruonan Ji (冀若楠), Zhipei Sun (孙志培), Shaowei Wang (王少伟)

**Affiliations:** ^1^ State Key Laboratory of Precision Spectroscopy, East China Normal University, Shanghai 200062, China.; ^2^ State Key Laboratory of Infrared Physics, Shanghai Institute of Technical Physics, Chinese Academy of Sciences, Shanghai 200083, China.; ^3^ Shanghai Research Center for Quantum Sciences, Shanghai 201315, China.; ^4^ QTF Centre of Excellence, Department of Electronics and Nanoengineering, Aalto University, Espoo 02150, Finland.; ^5^ Shanghai Key Laboratory of Multidimensional Information Processing, East China Normal University, Shanghai 200241, China.; ^6^ Collaborative Innovation Center of Extreme Optics, Shanxi University, Taiyuan, Shanxi 030006, China.

## Abstract

Tamm plasmon polariton (TPP) has unique localization and manipulability but cannot be excited with ultrathin metal films. We propose a strategy to realize generalized TPP (GTPP) in an ultrathin metal film as metainterface by introducing a low-loss dielectric Bragg reflector compensation layer, enabling effectively excitation of GTPP at its interface and achieving near-perfect absorption (~99.1%) at the resonant wavelength of 532 nanometers. This excitation has topological robustness, which fundamentally stems from its intrinsic tolerance to fabrication imperfections. A 14-channel narrowband absorbers chip based on GTPP has been fabricated. Compared to conventional structures without GTPP excitation, this device can lower the reverse saturable absorption threshold (~7.7 × 10^−5^ nanojoules per square micrometer) and increase the fluorescence intensity above the residual laser intensity to enhance the extinction capability by two orders of magnitude. These findings provide both evidence for applications of micro-nano photonic devices in fields such as laser elimination and offer insights for other scenarios.

## Teaser

A Bragg reflector stack enables near-perfect absorption of generalized Tamm plasmon polariton in ultrathin metals (<10 nm).

## INTRODUCTION

The optical Tamm state (OTS) is a unique optical surface state, akin to surface states in solid-state physics (*1*). Up to now, OTS has been demonstrated to exist at the interface between a photonic crystal and a material with a negative dielectric constant or another photonic crystal (*2*), including photonic crystal heterostructures (*3*–*5*) and metal-periodic dielectric structures (*6*, *7*). In these configurations, the optical field is confined near the interface between the metal and the periodic dielectric structure, forming localized optical modes. In 2007, Kaliteevski (*6*) theoretically predicted and experimentally observed Tamm plasmon polariton (TPP) at the interface of metal and periodic dielectric structures. Notably, TPP arises from the interaction between surface plasmon polaritons in the metal and the photonic bandgap of the photonic crystal, leading to a substantial enhancement of the electric field at the metal-photonic crystal interface. This unique localization and manipulability of TPP enable precise control through adjustments in the structure of the photonic crystal and the properties of the metal film (*8*–*12*). Recent advancements in incorporating active layers have enabled researchers to actively tune TPP under external electric (*13*) and magnetic fields (*14*), as well as temperature variations (*15*), highlighting their substantial potential for applications in perfect absorbers (*16*, *17*), sensors (*18*, *19*), hot electron photodetectors (*20*, *21*), and quantum lasers (*22*, *23*). When the metal film is sufficiently thin, the presence of abundant voids between the thin films may induce the generation of plasmon polaritons (*24*). It finds broad applications in sensors (*25*), plasmonics (*26*, *27*), and metamaterials (*28*–*30*) due to its low scattering and ohmic losses (*31*). Despite these advantages of TPP and ultrathin metal, research on exciting TPP in ultrathin metal remains largely unexplored. In particular, for metal thicknesses below 10 nm, the insufficient interaction between the metal and the photonic crystal cannot support TPP excitation.

Conventional metal–dielectric Bragg reflector (DBR) TPP systems typically use >10-nm metal films to meet two essential conditions (*32*): ω ≪ ω_p_ (where ω is the incident light frequency and ω_p_ is the plasma frequency of the metal) and *r*_DBR_ × *r*_metal_ = 1 (where *r*_DBR_ and *r*_metal_ are the reflection coefficients of the DBR and metal, respectively). So far, the thinnest metal thickness reported for TPP excitation is 10 nm, achieved by depositing an ultrathin metal film on a periodic dielectric structure, with successful application in surface-enhanced Raman spectroscopy (*33*). In addition, although composite metal nanoparticles can serve as an alternative to thin metal films for TPP excitation, their excitation frequency is fundamentally constrained by the plasmonic resonance of the nanoparticles (*34*). Meanwhile, the wavelength range of TPP systems remains bounded by the metal plasma frequency and fabrication-limited structural precision. To enable the practical applications of TPP in thin metals, it is essential to develop a universal and straightforward design strategy for TPP realization while minimizing the impact of fabrication defects and ensuring wavelength scalability.

Here, we present a strategy to achieve generalized TPP (GTPP) in an ultrathin metal film of metainterface that eliminates the need for complex processing or special patterns on the metal films to produce limited TPP. The proposed method allows for the innovative introduction of a low-loss DBR as a compensation layer adjacent to the ultrathin metal film, effectively converting the weak coupling between them into a stable surface state exhibiting GTPP characteristics. In detail, the GTPP mode undergoes metal-loss–driven symmetry breaking in its reflection coefficients, resulting in the formation of topologically protected phase singularity pairs while maintaining robustness against structural parameter variations to ensure stable operation. When combined with low-loss DBR as a compensation layer, our method offers a simple yet effective solution to a key challenge in TPP implementation. This approach significantly enhances light-matter interactions in ultrathin metal film while expanding both the theoretical framework and practical applications of TPP. The successful realization of GTPP in an ultrathin metal layer not only reduces noble material costs but also facilitates better integration with conventional semiconductor photonic components (*35*).

## RESULTS

### GTPP in ultrathin metal

**Fig. 1.**
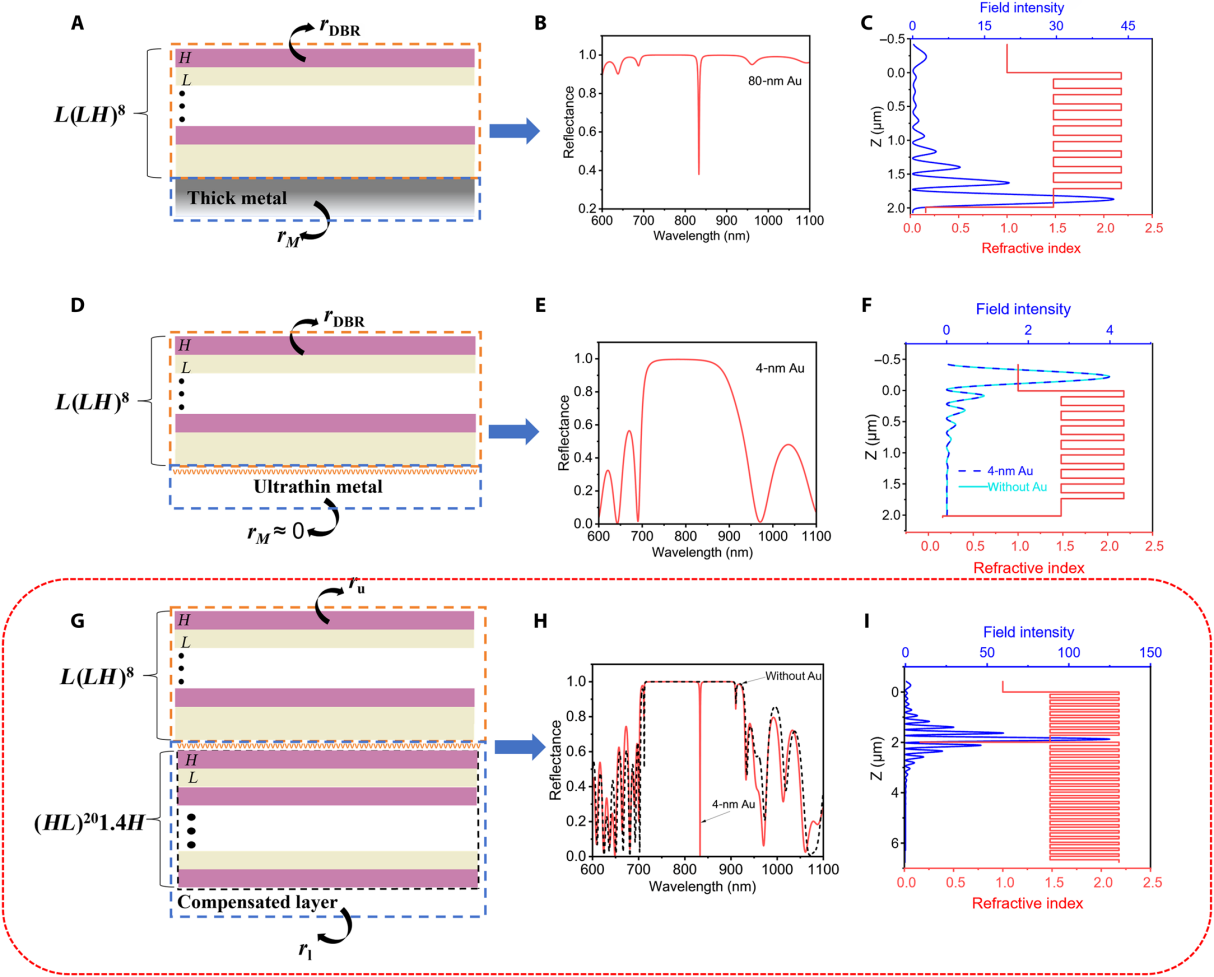
Design strategy for GTPP using ultrathin metal film. (**A**) Schematic of TPP excitation in a thick metal-DBR structure. (**B**) Reflection spectrum of TPP excitation with thick metal. (**C**) Refractive index profile (red solid line) and electric field distribution (blue solid line) at the resonant wavelength λ = 833 nm in (D). (**D**) Schematic of an ultrathin metal-DBR structure. (**E**) Reflection spectrum of the ultrathin metal-DBR structure. (**F**) Refractive index profile (red solid line) and electric field distribution (blue solid line) at λ = 833 nm in (E). (**G**) Introduction of a DBR compensation layer on the opposite side of the ultrathin metal. (**H**) Reflection spectrum showing GTPP excitation after adding the DBR compensation layer. (**I**) Refractive index profile (red solid line) and electric field distribution (blue solid line) at the resonant wavelength λ = 833 nm in (H).

OTS is a distinctive electromagnetic surface state that arises from the interaction between a metal and an one-dimensional crystal (or DBR), leading to the formation of a highly localized electric field mode at their interface (*5*). TPP demonstrates enhanced light-matter interactions owing to the strong field confinement at the interface, as shown in Fig. 1. In detail, Fig. 1A shows the conventional TPP excitation structure designed as Sub|*ML*(*LH*)^8^, where Sub denotes the substrate, *M* represents a 80-nm-thick Au layer, *L* is a 275-nm-thick SiO_2_ layer (refractive index *n* = 1.45), and *H* is a 94-nm-thick Ta_2_O_5_ layer (refractive index *n* = 2.13). According to the generation theory of TPP (*6*), when the metal layer thickness meets the requirement, the condition *r*_DBR_ *× r*_metal_ = 1 is satisfied, resulting in a sharp resonance peak in the transmission or reflection spectrum of the DBR, as shown in Fig. 1B. Using finite-difference time-domain (FDTD) simulations, we calculated the structure’s reflectivity spectrum. Besides, Fig. 1C reveals strong electric field confinement at the metal-DBR interface at resonance, which facilitates TPP generation through interaction with the metal film. However, when the metal film thickness decreases below a certain threshold (less than 10 nm), quantum size effects lead to material properties distinct from those of thicker metal (*36*). Under these conditions, the ultrathin metal’s reflection coefficient becomes substantially smaller than the DBR’s, violating the conventional TPP generation requirements. Consequently, the ultrathin metal-DBR’s reflection spectrum converges to match the DBR’s spectral characteristics, while the interfacial electric field distribution shifts from its characteristic exponential decay profile to one that closely mirrors the DBR’s intrinsic field pattern, as evidenced in Fig. 1 (D to F). These results indicate that an ultrathin metal film is incapable of supporting TPP excitation, as their reflection coefficient approaches zero, thereby violating the fundamental condition for Tamm plasmon generation. To address this issue, we propose introducing a low-loss compensation layer to form metainterface and effectively compensate for the reflection coefficient of the ultrathin metal, enabling the proper excitation of TPP.

As shown in Fig. 1G, we designed the structure as Sub|(*HL*)^20^1.4*HML*(*LH*)^8^, where (HL)^20^1.4*H* serves as the compensation layer, *M* represents a 4-nm-thick Au film, and *L*(*LH*)^8^ denotes the top DBR. The compensation layer effectively restores the reflection coefficient of the ultrathin metal, producing a distinct resonance peak near 833 nm in the reflection spectrum with near-zero reflectivity. Simultaneously, this configuration induces significant electric field enhancement at the ultrathin metal/top DBR interface, as demonstrated in Fig. 1 (H and I). Crucially, the compensation layer facilitates light localization at the metal-dielectric interface, enabling the excitation of GTPP. Unlike conventional OTSs arising from topological band inversion and photonic bandgap overlap in photonic crystal heterostructures (*37*), our DBR-based compensation approach provides the essential mechanism for GTPP realization in ultrathin metal systems.

**Fig. 2.**
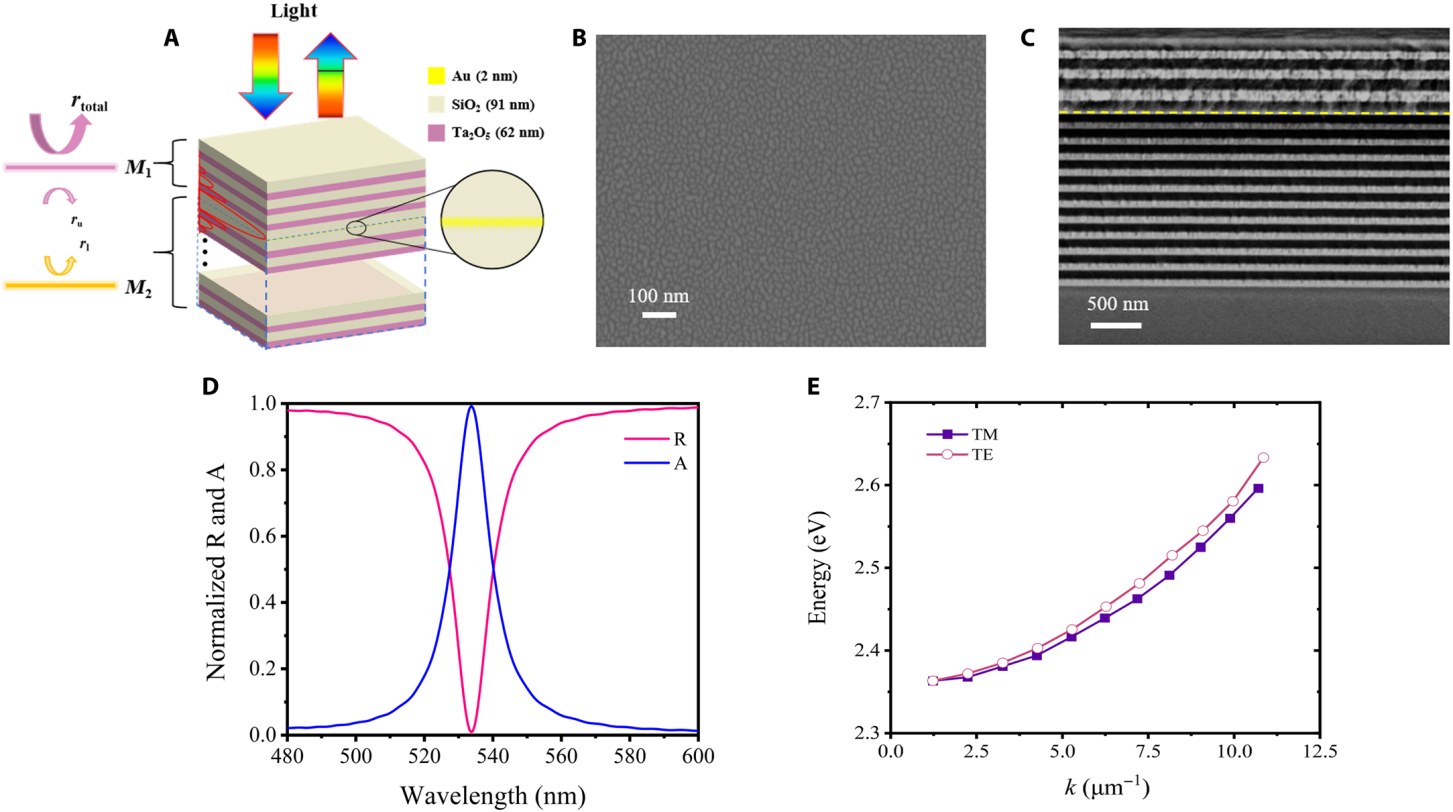
Schematic illustration and spectral characteristics of GTPP excitation in ultrathin metal structures. (**A**) Schematic of near-perfect light absorption through GTPP excitation in an ultrathin metal-dielectric structure, the red curve depicts the enhanced electric field distribution, and the detailed electromagnetic field distribution is shown in fig. S2. (**B**) Scanning electron microscopy (SEM) surface morphology of the fabricated 2-nm Au film. (**C**) Cross-sectional SEM image of the complete GTPP device structure. (**D**) Experimental reflection and absorption spectra of the GTPP device. R, reflection; A, absorption. (**E**) Calculated dispersion relations for GTPP modes under both transverse electric (TE) and transverse magnetic (TM) polarization.

For this purpose, we designed a simple structure to excite GTPP in ultrathin metal using a DBR compensation layer with the configuration Sub|(*HL*)^*m*2^*ML*(*HL*)^*m*1^ in addition, where *M* represents a 2-nm-thick Au film, *H* corresponds to a 62-nm-thick Ta_2_O_5_ layer, and *L* denotes a 91-nm-thick SiO_2_ layer. Compared to the 4-nm-thick metal film in Fig. 1G, the metal film thickness was designed to be 2 nm to investigate the influence of an even thinner metal layer on GTPP excitation and to validate the theory under more extreme conditions. Besides, the parameters *m*_1_ and *m*_2_ indicate the number of periods in the top DBR and compensation layer DBR, respectively, as illustrated in Fig. 2A. On the basis of the effective medium theory (*38*), the bottom DBR and the ultrathin metal film can be treated as an equivalent lower interface with the same reflection coefficient, while the top DBR serves as an equivalent upper interface with a reflection coefficient of $r_{u}$ . Together with the phase modulation layer between the ultrathin metal and the top DBR, these two reflection interfaces form a zero-reflection interface model, which can equivalently be interpreted as an asymmetric Fabry-Pérot (F-P) microcavity structure (*39*, *40*). According to the GTPP generation theory we proposed (see section S1), when $r_{u}=r_{l}^{*}$ , the reflection coefficient of the upper interface is equal and conjugate to that of the lower interface, the overall reflectivity of the structure becomes *R* = 0. To further suppress the transmission at the bottom of the asymmetric F-P microcavity and achieve zero transmittance for perfect absorption (*A* = 1 – *R* – *T*), the number of periods in the bottom DBR(*M*_2_) must satisfy the condition *m*_2_ > *m*_1_.

To validate the theoretical design, we fabricated a device with the multilayer structure Sub|(*HL*)^11^*ML*(*HL*)^3^, where *H* represents a 62-nm-thick Ta_2_O_5_ layer, *L* denotes a 91-nm-thick SiO_2_ layer, and *M* corresponds to a 2-nm-thick Au film, implementing the compensation mechanism described in the abovementioned. The surface morphology characterization revealed that the Au nanofilm exhibits an isolated island-like growth structure, as shown in Fig. 2B, with a root mean square (RMS) roughness of 1.085 nm. The RMS roughness of Au thin film when less than 10 nm is larger than those of larger than 10 nm, due to no continuous film forms yet. Notably, because of the lack of additional steps to enhance Au adhesion, the RMS value of this sample is higher than that of the ultrasmooth Au layer (*41*). In addition, cross-sectional scanning electron microscopy analysis in Fig. 2C shows the precise layer stacking with the critical Au interface position highlighted in yellow, demonstrating successful realization of the proposed asymmetric F-P microcavity structure for GTPP generation. During the experiment, the device was illuminated with a light source at a 6° incident angle, with reflection spectra measured using a visible–near-infrared spectrophotometer (Agilent Cary 7000). As shown in Fig. 2D, the excitation of GTPP achieves near-perfect absorption (*A* = 0.991) at a resonance wavelength of 532 nm. Given that the top DBR consists of 3 pairs and the bottom DBR comprises 11 pairs, this exceptional absorption arises from the narrowband filtering mechanism of the asymmetric F-P microcavity, which induces strongly localized field enhancement in the 2-nm Au nanofilm. Notably, when | $r_{l}$| = |$r_{u}$| = 1, the formulas $r_{u}=r_{l}^{*}$ and $r_{l}\;$ × $r_{u}$ = 1 are equivalent, demonstrating that traditional TPP excitation is a special case within our generalized GTPP framework. These results confirm that the zero-reflection interface theory $r_{u}=r_{l}^{*}$ provides a universal and experimentally verifiable approach for realizing GTPP in ultrathin metal-dielectric systems. By selecting appropriate structural parameters, such as the number of periods *m*_1_ and *m*_2_ in the top and bottom DBRs, to satisfy the zero-reflection condition, GTPP can be excited in arbitrary ultrathin metal (see section S2 for the effects of different metals). Figure 2E shows the dispersion relationship of GTPP energy as a function of the in-plane wave vector *k* for TE and TM modes. The dispersion curve obtained from angle-resolved measurements exhibits a parabolic shape, consistent with the linear dispersion of TPP described in the reference (*6*). Consequently, this dispersion relationship can be fitted to extract an effective mass of *m* = 2.66 × 10^−5^ *m*_0_ for the TM mode, where *m*_0_ is the free electron mass (the “Spectra of GTPP devices as a function of the incident angle” section is shown in section S3).

### Tunable topological GTPP

**Fig. 3.**
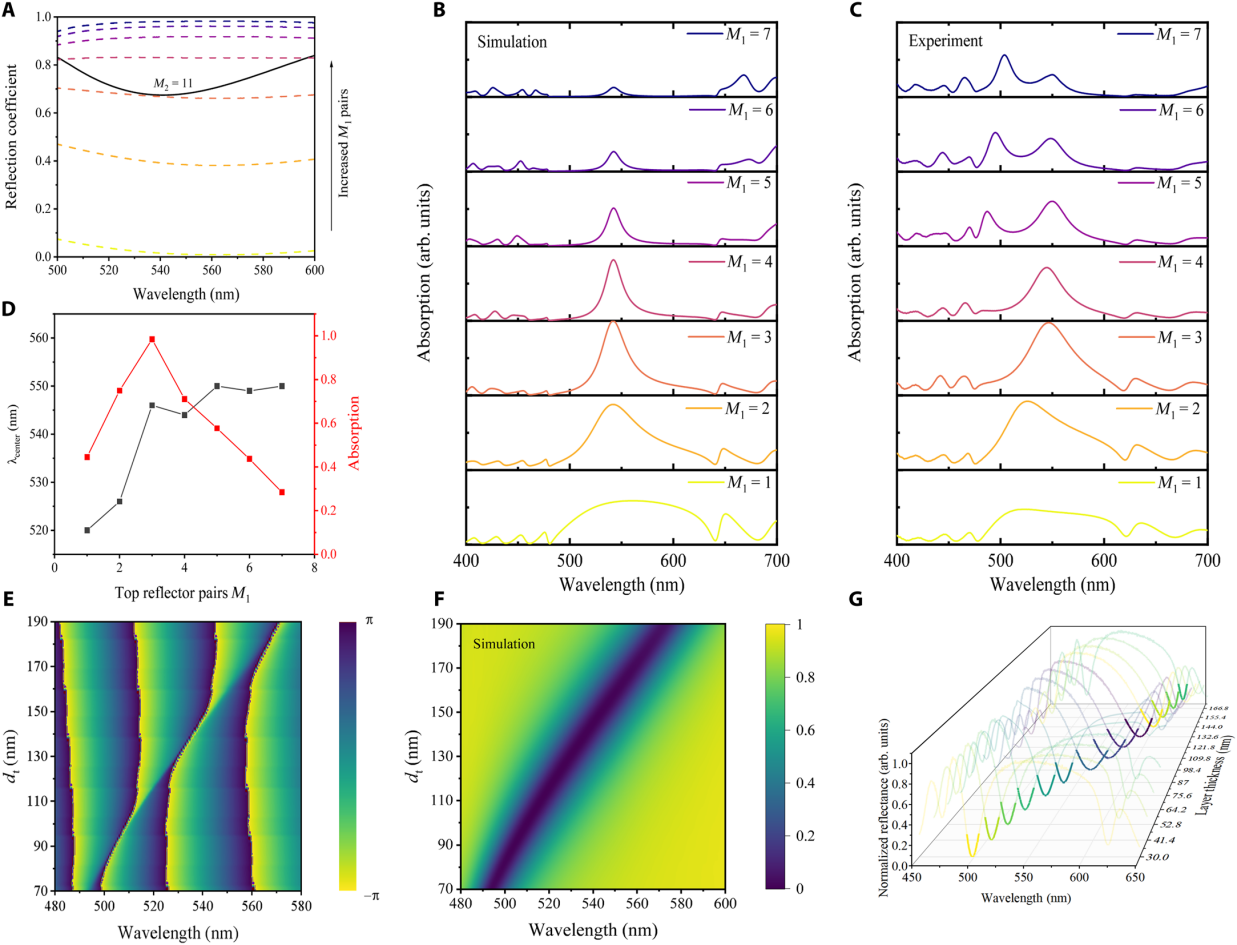
Tunability of GTPP characteristics through structural engineering. (**A**) Calculated reflection coefficient for the 2-nm metal film with fixed bottom DBR pairs (*m*_2_ = 11) as a function of increasing top DBR pairs (*m*_1_). The black reference curve represents the base reflection coefficient at the ultrathin metal/DBR compensation layer interface. (**B**) Numerically simulated absorption spectra showing the evolution of GTPP resonances with different numbers of top DBR pairs. (**C**) Experimentally measured absorption spectra of fabricated GTPP devices with varying top DBR pairs. (**D**) Quantitative analysis of absorption peak positions and corresponding absorption efficiencies for different top DBR pairs. (**E**) Calculated reflection phase variation at the metal–top DBR interface as a function of phase-matching layer thickness (from 70 to 190 nm). (**F**) Simulated reflection spectra showing the effect of phase-matching layer thickness variation. (**G**) Normalized reflection spectra of the fabricated 14-channel GTPP device array; phase-matching layer thickness (*d*_t_ = 30 to 180 nm, and the spacing is ~11.4 nm).

The realization of GTPP in ultrathin metal requires careful control of two critical parameters: the number of periods in the top DBR (*m*_1_), which determines the upper interface’s reflection coefficient, and the phase modulation layer thickness that governs the reflection phase. As shown in Fig. 3A, increasing *m*_1_ from 1 to 7 periods leads to a systematic enhancement of the reflection coefficient at 532 nm, with values rising progressively from 0.02 to 0.97. The black reference curve in the figure represents the base reflection coefficient of the interface formed by the ultrathin metal and DBR compensation layer, providing an essential baseline for evaluating the parameter optimization effects. When *m_1_* = 3, the reflection coefficients of the base interface and the top DBR are equal at 532 nm, and the reflection phases exhibit mutually opposite values (see section S4 for details), validating the aforementioned relationship $r_{u}=r_{l}^{*}$ . Combined with Fig. 3B, it can be observed that as *m*_1_ increases, the absorption of the GTPP device first increases and then decreases, because the reflection coefficient of the upper interface is modulated by *m*_1_, and maximum absorption occurs only when the reflection coefficients of the upper and lower interfaces are matched. As shown in Fig. 3C, the measured absorption spectra under different *m*_1_ values demonstrate good agreement with the simulation results, confirming the absorption peak intensities. Notably, when *m*_1_ exceeds 5, distinct secondary peaks emerge in experimental results, deviating from simulation outcomes. This discrepancy primarily comes from accumulated deviations between the actual films’ thickness and designed ones. Ultimately, these accumulating thickness variations cause misalignment between experimental and simulated data when *m*_1_ exceeds 5 (see section S5 for the details). Moreover, Fig. 3D reveals that when *m*_1_ = 3 and *m*_2_ = 11, the GTPP device achieves maximum narrowband absorption enhanced by plasmonic effects. This approach can be generalized to ultrathin metal by designing equivalent reflection coefficients to match the upper and lower interfaces, thereby exciting GTPP and enabling narrowband absorption.

In Fig. 3E, it shows that the reflection phase of the structure undergoes a zero-crossing jump as the thickness (*d*_t_) of the phase modulation layer increases from 70 to 190 nm, with the phase jump gradually red shifting in correspondence with the absorption peak. Under normal incidence conditions, increasing *d*_t_ from 70 to 190 nm induces a redshift of the resonance peak from 495 to 569 nm, corresponding to a total wavelength shift of 74 nm, as shown in Fig. 3F. It is evident that the zero-crossing jump coincides with the absorption peak shift. To further validate this tunability, we fabricated a 14-channel GTPP filter on chip using combinatorial lithography, incorporating phase modulation layers of varying thicknesses. The measured reflection spectra reveal that as the layer thickness increases from 30 to 180 nm and the GTPP peak shifts from 498 to 618 nm, fully spanning the DBR stopband, as shown in Fig. 3G. These results demonstrate that the absorption peak position can be precisely controlled by adjusting the thickness of the phase modulation layer.

**Fig. 4.**
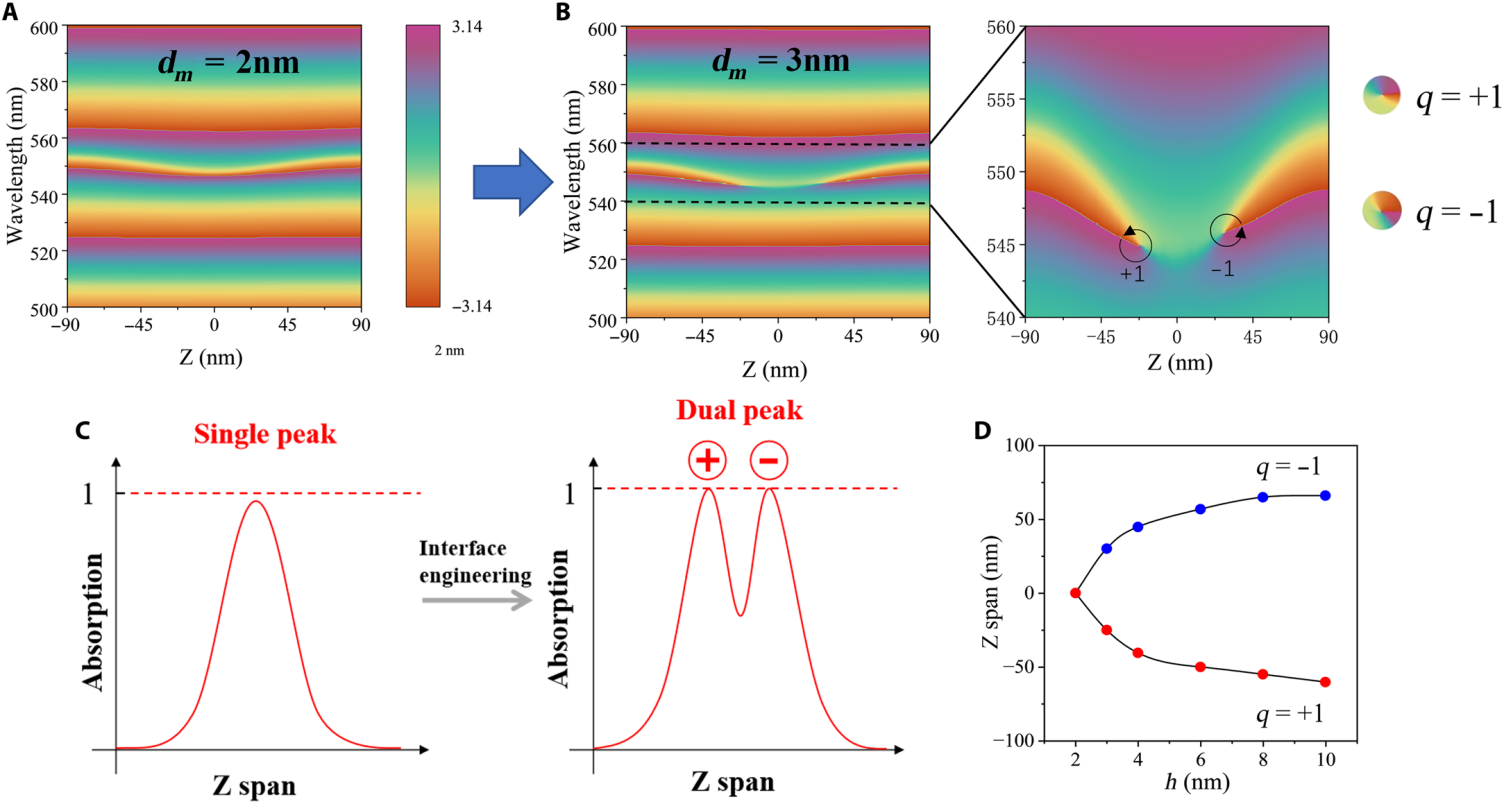
Topological characteristics of SPSs in GTPP systems (simulation). (**A**) Reflection phase distribution in λ-p space for a 2-nm-thick metal. (**B**) Evolution of SPSs in λ-p space with increasing metal thickness (3-nm case). (**C**) Dual-peak perfect absorption (PA) mechanism based on topological charge evolution. Left: Single absorption peak (no PA) with annihilated SPSs. Right: Dual PA peaks accompanied by generation of *q* = ±1 topological charge pair. (**D**) Evolution pathways of topological phase singularity charges as functions of metal thickness in dielectric microcavities.

In practical device fabrication, manufacturing tolerances are inevitable, making robustness against parameter variations particularly crucial. Notably, the excitation of GTPP demonstrates exceptional robustness to metallic losses. This property is analytically explained by examining the topological characteristics of GTPP, inducing absorption peaks and spectral phase singularities (SPSs) at ultrathin metal-dielectric interfaces. Besides, even when the thickness of the metal layer changes, leading to a change in the reflectance, topological phase singularities still exist, corresponding to the excitation of GTPP and the existence of perfect absorption points. The underlying mechanism further reveals GTPP’s intrinsic tolerance to fabrication imperfections. SPSs emerge at zero-reflection or zero-transmission points in the spectral response. When these singularities are encircled in the spectral-parameter phase space, they yield integer topological invariants. The topological phase singularity charge is defined as q=∮dφ/2π,q∈Z , where φ represents the reflection phase (*42*). In position-wavelength space, pairs of these topological charges correspond to dual perfect absorption points within the metal thin film. Figure 4A demonstrates that a 2-nm Au film placed at the microcavity center generates a single near-perfect absorption point under GTPP resonance, accompanied by a continuous phase variation (topological charge *q* = 0). As metallic loss increases, this absorption point splits into two topologically protected perfect absorption points, as shown in Fig. 4B. For a thicker metal film, the phase map reveals two counterrotating phase vortices (*q* = +1 and *q* = −1), conserving the total topological charge. Figure 4C illustrates this evolution of dual absorption points, driven by topological charge dynamics. The underlying mechanism stems from the reduced effective reflection coefficient at the Au-DBR interface with increasing metal thickness. To sustain zero reflection, the Au layer must shift further to compensate for the diminished reflectivity, adapting to the modified boundary conditions. The corresponding evolution of SPSs is depicted in Fig. 4D, with the reflection phase distribution in wavelength-position (λ-p) space for varying metal thicknesses provided in section S5.

### Applications of GTPP

**Fig. 5.**
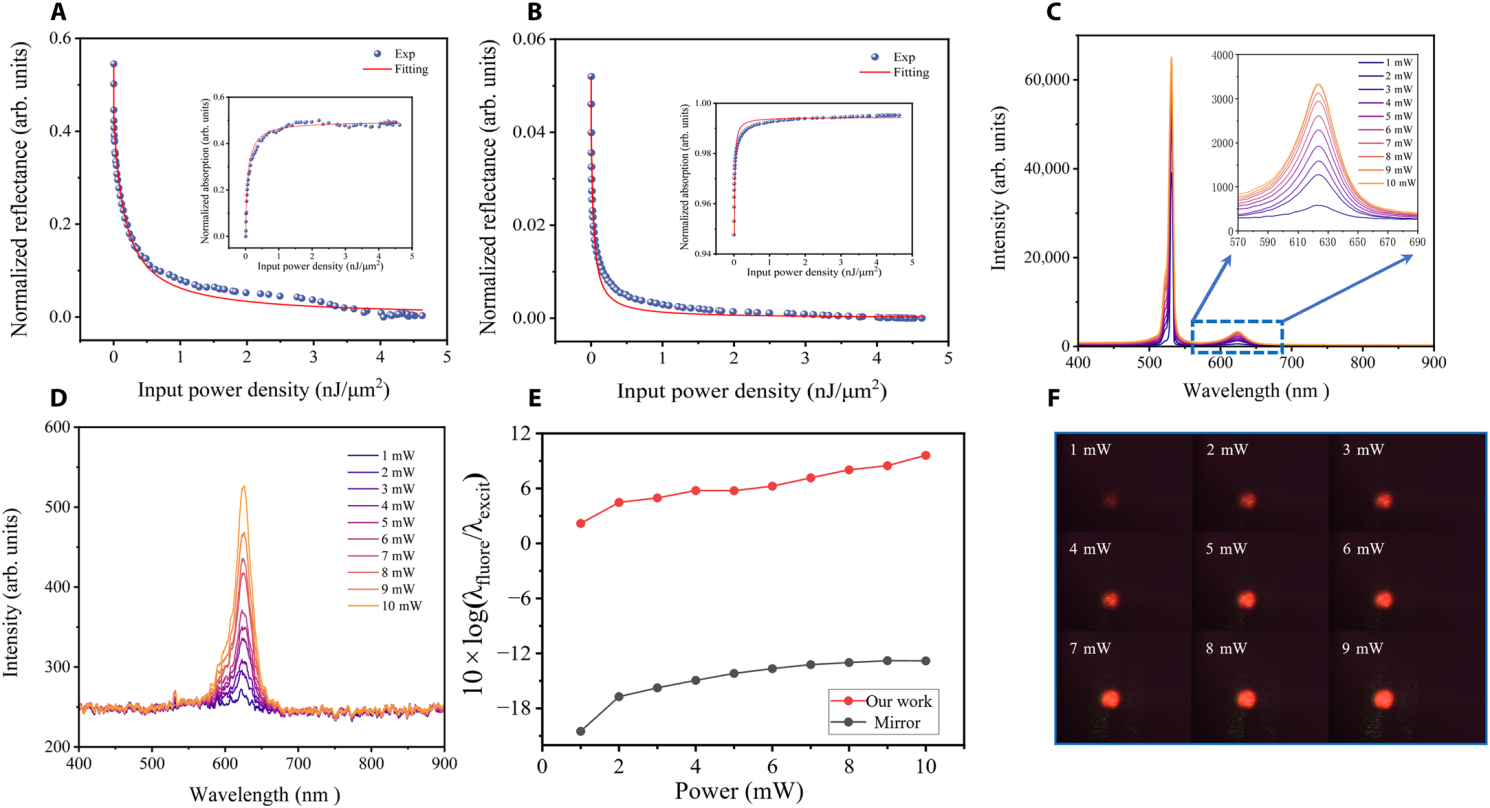
Nonlinear absorption characteristics and stray laser suppression using GTPP devices. (**A**) Normalized reflectance of a 2-nm Au ultrathin film under increasing incident power density, showing RSA behavior. The reflectance decreases with higher power densities, while the normalized absorption (inset) increases and eventually saturates. The RSA threshold is measured at 9.6 × 10^−3^ nJ/μm^2^. (**B**) GTPP-enhanced RSA response, exhibiting a significantly lower threshold (7.7 × 10^−5^ nJ/μm^2^) compared to bare Au film. The reflectance drop is more pronounced, and the absorption (inset) saturates at lower power densities, demonstrating enhanced nonlinear plasmonic effects. (**C**) Fluorescence spectrum of CdSe quantum dots excited by a 532-nm laser, showing superstrong residual laser interference overwhelming the 625-nm emission signal (see the “Spectroscopy measurements” section). (**D**) With the GTPP device replacing the conventional mirror, the 532-nm laser line is completely suppressed. (**E**) Comparison of extinction performance between a metal mirror and the GTPP device across varying laser intensities (1 to 10 mW). fluore, fluorescence; excit, excitation. (**F**) Fluorescence imaging of CdSe quantum dots under 532 nm excitation. The GTPP-processed images show brighter emission, uniform intensity distribution, and no laser-induced artifacts. Exp, experiment.

Because their near-perfect absorption at the resonant wavelength, we investigated the nonlinear absorption characteristics of GTPP-based narrowband absorbers and explored their applicability for residual laser suppression. To evaluate their optical response under varying incident intensities, we first performed reflection-mode power-scan measurements on the fabricated samples, including both Au nanofilm on glass substrates and GTPP-integrated devices, analyzing their reflectance and transmittance characteristics. Figure 5A displays the normalized reflection curve of the Au nanofilm after subtracting substrate and baseline contributions, with the inset presenting the normalized absorption curve (blue scatter: experimental data). As the incident energy density increases, the reflectance of the Au nanofilm progressively decreases until reaching saturation, accompanied by a concurrent rise in absorption into the saturated regime. This trend is consistent with the definition of reverse saturable absorption (RSA). Similarly, the GTPP device exhibits RSA in Fig. 5B but achieves saturation at a substantially lower energy density compared to the Au nanofilm. To quantitatively analyze this effect, we fitted the normalized reflectance/transmittance spectra using the model (*43*): $R=1-T_{0}T_{A}/\left(1+E_{\text{in}}/E_{\text{sat}}\right)$ , where $E_{\text{in}}$ is the incident energy, $E_{\text{sat}}$ represents the saturation fluence, $T_{A}$ denotes the aperture transmittance, and $T_{0}$ is the lens transmittance (with $T_{0}T_{A}$ treated as a constant). The saturation threshold energy is defined as $E_{t}$ = $\gamma E_{\text{sat}}$ , where γ = 1 corresponds to the threshold at half the saturation fluence.

The fitted curves [blue lines in Fig. 5 (A and B)] reveal an RSA threshold of 9.6 × 10^−3^ nJ/μm^2^ for the Au nanofilm, while the GTPP device demonstrates a marked lower threshold of 7.7 × 10^−5^ nJ/μm^2^, representing a two–order-of-magnitude reduction. This decrease in the RSA threshold underscores the asymmetric F-P cavity’s exceptional ability to enhance nonlinear plasmonic absorption. These results establish an effective design strategy for developing high-performance, ultrasensitive nonlinear plasmonic devices with significantly improved efficiency. The exceptional nonlinear RSA performance and ultralow RSA threshold of our GTPP-based narrowband absorbers demonstrate outstanding potential for suppressing residual laser interference in fluorescence spectroscopy. To validate this application, we excited CdSe quantum dots using a 532-nm laser with tunable intensity (1 to 10 mW) and collected the resulting fluorescence spectra through an optical fiber spectrometer via the reflection measurement path shown in Fig. 5C. Initial measurements revealed strong residual laser signals dominating the fluorescence spectrum (inset: magnified fluorescence signal). For suppression, we implemented a GTPP device nominally resonant at 550 nm. By tuning the incident angle to 26° under *s*-polarization, we achieved a redshifted resonance at 532 nm, replacing the conventional mirror. As shown in Fig. 5D, the GTPP device effectively absorbed the 532-nm residual laser across all tested intensities, successfully eliminating interference with weak fluorescence signals. The quantitative comparison in Fig. 5E indicates that the GTPP device increases the fluorescence intensity above the residual laser intensity and enhances the extinction capability by two orders of magnitude compared to the conventional mirror, showing additional improvement with increasing laser intensity. Figure 5F presents fluorescence imaging under varying laser powers, demonstrating brighter and sharper images without laser-induced distortion and confirming the device’s robustness for high-power strong-signal applications.

## DISCUSSION

In summary, we present a mechanism for exciting GTPP at ultrathin metal-dielectric multilayer interfaces. The proposed metainterface structure incorporates DBR beneath the ultrathin Au nanofilm to compensate for the effective reflection coefficient. Through careful optimization of both the number of top DBR pairs and the phase-matching layer thickness, we achieve precise control over the reflection coefficient and phase at the dielectric interface. In addition, a systematic investigation of SPSs and conservation of topological phase charges during GTPP excitation reveals exceptional robustness against metallic losses. Using combined photolithography and etching techniques, we demonstrate precise control of the phase-matching layer thickness, enabling tunable positioning of GTPP-induced absorption peaks at the Au-DBR interface. The GTPP-enhanced structure exhibits significantly improved nonlinear reverse RSA, achieving an ultralow RSA threshold of 7.7 × 10^−5^ nJ/μm^2^ and representing a two–order-of-magnitude reduction compared to without DBR structures. Last, we successfully implemented this enhanced nonlinear plasmonic absorption for residual laser suppression in fluorescence spectroscopy, demonstrating superior extinction performance that scales favorably with increasing laser intensity. These results establish a framework for manipulating nonlinear plasmonic effects, with notable implications for developing advanced nanophotonic devices, including optical sensors, nano lasers, and precision measurement systems.

## MATERIALS AND METHODS

### Device fabrication

The GTPP device with an (*HL*)^11^*ML*(*HL*)^3^ multilayer structure was fabricated as follows: The K9 glass substrate was ultrasonically cleaned in acetone and isopropanol, rinsed with deionized water, and dried with a nitrogen gun. A bottom DBR was deposited using an electron beam evaporation system (Leybold, ARES 1110), with alternating Ta_2_O_5_ and SiO_2_ layers. A 2-nm Au layer was then sputtered using a magnetron sputtering deposition system (K. J. Lesker, PRO Line PVD 75). For the phase modulation layer, a SiO_2_ film was deposited by electron beam evaporation. To fabricate the 14-channel filter array on a single substrate, photolithography was performed using ML3 Aligner system, followed by inductively coupled plasma etching (Oxford, PlasmaPro System 100) to create stepped cavity structures. This lithography-etching cycle was repeated four times to achieve 14 discrete thicknesses. Last, three DBR pairs (Ta_2_O_5_/SiO_2_) were deposited by electron beam evaporation to complete the device, with each channel measuring an area of 250 μm by 250 μm. The detailed fabrication parameters and key parameters of the experimental equipment are provided in the sections S7 and S8.

### Spectroscopy measurements

Reflection-mode power-scan measurement optical path (as illustrated in fig. S7): A nanosecond pulsed laser with 532 nm excitation wavelength and 100 Hz repetition rate serves as the incident light source. The incident laser is converted to linearly polarized light through a polarizer and then transformed into circularly polarized light via a half-wave plate. After passing through another polarizer, the light intensity attenuation varies with changes in the incident polarization state. By adjusting the angle between the polarizer and the half-wave plate’s optical axis using a motorized rotation stage, the incident light intensity can be precisely controlled. Two photodetectors are used to simultaneously measure the sample’s transmitted and reflected signals.

Microscopic fluorescence detection system optical path (as illustrated in fig. S8): The excitation source is a 532-nm continuous-wave laser, which is expanded by a beam expander to produce a 5-mm-diameter collimated beam. This beam is then focused onto CdSe quantum dots via a lens, generating fluorescence signals at 625-nm wavelength. The emitted fluorescence is reflected by a mirror and collected by a fiber-optic spectrometer.

### Numerical simulations

The numerical simulations were performed using the FDTD method. The optical constants of Ta_2_O_5_, SiO_2_, and Au were experimentally determined before simulation. We used the commercial FDTD Solutions software, implementing periodic boundary conditions along the *x* directions, perfectly matched layer boundary conditions along the *y* direction in two-dimensional simulations. The light source is set as a broadband fixed-angle plane wave.

## Supplementary Materials

### This PDF file includes:

Supplementary Text Figs. S1 to S8 References

## References

[1] H. Ohno, E. E. Mendez, A. Alexandrou, J. M. Hong, Tamm states in superlattices. Surf. Sci. 267, 161–165 (1992).

[2] A. M. Merzlikin, M. Inoue, A. P. Vinogradov, A. V. Dorofeenko, A. B. Granovsky, A. A. Lisyansky, Tamm state at one-dimensional photonic crystals. J. Magn. Soc. Jpn. 30, 616–619 (2006).

[3] J.-Y. Guo, Y. Sun, H.-Q. Li, Y.-W. Zhang, H. Chen, Optical Tamm states in dielectric photonic crystal heterostructure. Chin. Phys. Lett. 25, 2093–2096 (2008).

[4] T. Goto, A. V. Dorofeenko, A. M. Merzlikin, A. V. Baryshev, A. P. Vinogradov, M. Inoue, A. A. Lisyansky, A. B. Granovsky, Optical tamm states in one-dimensional magnetophotonic structures. Phys. Rev. Lett. 101, 113902 (2008). 18851281 10.1103/PhysRevLett.101.113902

[5] J. Guo, Y. Sun, Y. Zhang, H. Li, H. Jiang, H. Chen, Experimental investigation of interface states in photonic crystal heterostructures. Phys. Rev. E 78, 026607 (2008). 10.1103/PhysRevE.78.026607 18850962

[6] M. Kaliteevski, I. Iorsh, S. Brand, R. A. Abram, J. M. Chamberlain, A. V. Kavokin, I. A. Shelykh, Tamm plasmon-polaritons: Possible electromagnetic states at the interface of a metal and a dielectric Bragg mirror. Phys. Rev. B 76, 165415 (2007).

[7] M. E. Sasin, R. P. Seisyan, M. A. Kalitteevski, S. Brand, R. A. Abram, J. M. Chamberlain, A. Y. Egorov, A. P. Vasil'ev, V. S. Mikhrin, A. V. Kavokin, Tamm plasmon polaritons: Slow and spatially compact light. Appl. Phys. Lett. 92, 251112 (2008).

[8] S. H. Tsang, S. F. Yu, X. F. Li, H. Y. Yang, H. K. Liang, Observation of Tamm plasmon polaritons in visible regime from ZnO/Al_2_O_3_ distributed Bragg reflector - Ag interface. Opt. Commun. 284, 1890–1892 (2011).

[9] Y.-t. Fang, L.-k. Chen, N. Zhu, J. Zhou, Tamm states of one-dimensional metal-dielectric photonic crystal. IET Optoelectron. 7, 9–13 (2013).

[10] Y. Chen, D. Zhang, L. Zhu, Q. Fu, R. Wang, P. Wang, H. Ming, R. Badugu, J. R. Lakowicz, Effect of metal film thickness on Tamm plasmon-coupled emission. Phys. Chem. Chem. Phys. 16, 25523–25530 (2014). 25349013 10.1039/c4cp04031g PMC4438750

[11] H. Zhou, G. Yang, K. Wang, H. Long, P. Lu, Multiple optical Tamm states at a metal-dielectric mirror interface. Opt. Lett. 35, 4112–4114 (2010). 21165107 10.1364/OL.35.004112

[12] C.-Y. Chang, Y.-H. Chen, Y.-L. Tsai, H.-C. Kuo, K.-P. Chen, Tunability and optimization of coupling efficiency in tamm plasmon modes. IEEE J. Sel. Top. Quantun Electron. 21, 262–267 (2015).

[13] L. Li, H. Zhao, J. Zhang, Electrically tuning reflection of graphene-based Tamm plasmon polariton structures at 1550 nm. Appl. Phys. Lett. 111, 083504 (2017).

[14] P. S. Pankin, V. S. Sutormin, V. A. Gunyakov, F. V. Zelenov, I. A. Tambasov, A. N. Masyugin, M. N. Volochaev, F. A. Baron, K. P. Chen, V. Y. Zyryanov, S. Y. Vetrov, I. V. Timofeev, Experimental implementation of tunable hybrid Tamm-microcavity modes. Appl. Phys. Lett. 119, 161107 (2021).

[15] Y.-t. Fang, Y.-x. Ni, H.-q. He, J.-x. Hu, Effect of hybrid state of surface plasmon-polaritons, magnetic defect mode and optical Tamm state on nonreciprocal propagation. Opt. Commun. 320, 99–104 (2014).

[16] G. Lu, K. Zhang, Y. Zhao, L. Zhang, Z. Shang, H. Zhou, C. Diao, X. Zhou, Perfect optical absorbers by all-dielectric photonic crystal/metal heterostructures due to optical Tamm state. Nanomaterials 11, 3447 (2021). 34947796 10.3390/nano11123447 PMC8709068

[17] Y. Gong, X. Liu, H. Lu, L. Wang, G. Wang, Perfect absorber supported by optical Tamm states in plasmonic waveguide. Opt. Express 19, 18393–18398 (2011). 21935207 10.1364/OE.19.018393

[18] R. Das, T. Srivastava, R. Jha, Tamm-plasmon and surface-plasmon hybrid-mode based refractometry in photonic bandgap structures. Opt. Lett. 39, 896–899 (2014). 24562235 10.1364/OL.39.000896

[19] W. L. Zhang, F. Wang, Y. J. Rao, Y. Jiang, Novel sensing concept based on optical Tamm plasmon. Opt. Express 22, 14524–14529 (2014). 24977548 10.1364/OE.22.014524

[20] C. Zhang, K. Wu, V. Giannini, X. Li, Planar hot-electron photodetection with Tamm plasmons. ACS Nano 11, 1719–1727 (2017). 28117569 10.1021/acsnano.6b07578

[21] W. Liang, Z. Xiao, H. Xu, H. Deng, H. Li, W. Chen, Z. Liu, Y. Long, Ultranarrow-bandwidth planar hot electron photodetector based on coupled dual Tamm plasmons. Opt. Express 28, 31330–31344 (2020). 33115108 10.1364/OE.400258

[22] C. Symonds, G. Lheureux, J. P. Hugonin, J. J. Greffet, J. Laverdant, G. Brucoli, A. Lemaitre, P. Senellart, J. Bellessa, Confined Tamm plasmon lasers. Nano Lett. 13, 3179–3184 (2013). 23777399 10.1021/nl401210b

[23] G. Lheureux, S. Azzini, C. Symonds, P. Senellart, A. Lemaître, C. Sauvan, J.-P. Hugonin, J.-J. Greffet, J. Bellessa, Polarization-controlled confined Tamm plasmon lasers. ACS Photonics 2, 842–848 (2015).

[24] P. Zhao, W. T. Su, R. Wang, X. F. Xu, F. S. Zhang, Properties of thin silver films with different thickness. Phys. E 41, 387–390 (2009).

[25] H. Liu, B. Wang, E. S. P. Leong, P. Yang, Y. Zong, G. Si, J. Teng, S. A. Maier, Enhanced surface plasmon resonance on a smooth silver film with a seed growth layer. ACS Nano 4, 3139–3146 (2010). 20515054 10.1021/nn100466p

[26] M. Mayy, G. Zhu, E. Mayy, A. Webb, M. A. Noginov, Low temperature studies of surface plasmon polaritons in silver films. J. Appl. Phys. 111, 094103 (2012).

[27] L. Ke, S. C. Lai, H. Liu, C. K. N. Peh, B. Wang, J. H. Teng, Ultrasmooth silver thin film on PEDOT:PSS nucleation layer for extended surface plasmon propagation. ACS Appl. Mater. Interfaces 4, 1247–1253 (2012). 22339782 10.1021/am201391f

[28] Y. Zhang, Z. Li, F. Cao, T. Tang, T. Ding, Ultra-wide stopband polarization narrow band filter combining optical Tamm state engineering and grating structure. J. Nanophotonics 18, 046006 (2024).

[29] J. Zhang, L. Long, Y. Wu, H. Ye, M. Liu, Metafilms for visible and infrared compatible camouflage of high-temperature targets. Mater Res Express 10, 036402 (2023).

[30] C. C. Chang, T. Y. Chen, T. W. Lin, J. F. Leng, K. Tamada, Y. J. Lee, Flexible and ultranarrow transmissive color filters by simultaneous excitations of triple resonant eigenmodes in hybrid metallic-optical tamm state devices. ACS Photonics 8, 540–549 (2021).

[31] A. Ciesielski, L. Skowronski, M. Trzcinski, T. Szoplik, Controlling the optical parameters of self-assembled silver films with wetting layers and annealing. Appl. Surf. Sci. 421, 349–356 (2017).

[32] C. Kar, S. Jena, D. V. Udupa, K. D. Rao, Tamm plasmon polariton in planar structures: A brief overview and applications. Opt. Laser Technol. 159, 108928 (2023).

[33] K. V. Sreekanth, J. Perumal, U. S. Dinish, P. Prabhathan, Y. Liu, R. Singh, M. Olivo, J. Teng, Tunable Tamm plasmon cavity as a scalable biosensing platform for surface enhanced resonance Raman spectroscopy. Nat. Commun. 14, 7085 (2023). 37925522 10.1038/s41467-023-42854-7 PMC10625559

[34] S. Y. Vetrov, P. S. Pankin, I. V. Timofeev, The optical Tamm states at the interface between a photonic crystal and a nanocomposite containing core-shell particles. J. Optics 18, 065106 (2016).

[35] C. Zhang, Q.-Y. Huang, Q. Cui, C. Ji, Z. Zhang, X. Chen, T. George, S. Zhao, L. J. Guo, High-performance large-scale flexible optoelectronics using ultrathin silver films with tunable properties. ACS Appl. Mater. Interfaces 11, 27216–27225 (2019). 31282144 10.1021/acsami.9b08289

[36] K. M. Tsysar, E. M. Smelova, A. M. Saletsky, V. G. Andreev, Quantum size effect in conductive properties of silver nanofilms. Thin Solid Films 710, 138263 (2020).

[37] K. Xu, J. Zhang, B. Yang, F. Wu, C. Yin, Non-reciprocal optical Tamm state in a photonic crystal heterojunction containing Weyl semimetals. Phys. B. Condens. Matter 691, 416329 (2024).

[38] B. Abeles, J. I. Gittleman, Composite material films: Optical properties and applications. Appl. Optics 15, 2328–2332 (1976). 10.1364/AO.15.002328 20165394

[39] X. Guan, Q. Liu, C. Li, Z. Yin, J. Wu, P. Yu, W. Lu, S. Wang, Generalized Fano resonance theory based on Fabry-Perot cavity. J. Phys. D. Appl. Phys. 57, 135102 (2024).

[40] Z. Xuan, J. Li, Q. Liu, F. Yi, S. Wang, W. Lu, Artificial structural colors and applications. The Innovation 2, 100081 (2021). 34557736 10.1016/j.xinn.2021.100081 PMC8454771

[41] L. Leandro, R. Malureanu, N. Rozlosnik, L. Lavrinenko, Ultrathin, ultrasmooth gold layer on dielectrics without the use of additional metallic adhesion layers. ACS Appl. Mater. Interfaces 7, 5797–5802 (2015). 25723253 10.1021/am508681u

[42] M. Liu, W. Chen, G. Hu, S. Fan, D. N. Christodoulides, C. Zhao, C.-W. Qiu, Spectral phase singularity and topological behavior in perfect absorption. Phys. Rev. B 107, L241403 (2023).

[43] D. Vincent, Optical limiting threshold in carbon suspensions and reverse saturable absorber materials. Appl. Optics 40, 6646–6653 (2001). 10.1364/ao.40.006646 18364974

[44] H. A. Macleod, *Thin-Film Optical Filters* (CRC Press, ed. 3, 2001).

[45] Q. Liu, X. Zhao, C. Li, X. Zhou, Y. Chen, S. Wang, W. Lu, Coupled Tamm plasmon polaritons induced narrow bandpass filter with ultra-wide stopband. Nano Res 15, 4563–4568 (2022).

[46] D. I. Yakubovsky, Y. V. Stebunov, R. V. Kirtaev, G. A. Ermolaev, M. S. Mironov, S. M. Novikov, A. V. Arsenin, V. S. Volkov, Ultrathin and ultrasmooth gold films on monolayer MoS_2_. Adv. Mater. Interfaces 6, 1900196 (2019).

[47] J. Gong, R. Dai, Z. Wang, Z. Zhang, Thickness dispersion of surface plasmon of ag nano-thin films: Determination by ellipsometry iterated with transmittance method. Sci. Rep. 5, 9279 (2015). 25797217 10.1038/srep09279 PMC4369689

[48] S. Gao, J. Lian, P. Song, P. Li, Z. Ma, X. Wang, S. Wu, “Study on optical constant of ultrathin aluminum films deposited by molecular beam epitaxy,” in *2011 Symposium on Photonics and Optoelectronics* (IEEE, 2011), pp. 4–4.

