# Supplementary material for: Excitation of Tamm plasmon polariton in ultrathin metals
Source: Sci Adv. 2025 Oct 22;11(43):eadz0106. doi: 10.1126/sciadv.adz0106 (PMC12542941; doi:10.1126/sciadv.adz0106)
Supplement: Supplementary file 1 — Supplementary Text Figs. S1 to S8 References [file sciadv.adz0106_sm.pdf]

Science Advances
AAAS

Supplementary Materials for

Excitation of Tamm plasmon polariton in ultrathin metals

Jiangwei Zhang *et al.*

Corresponding author: Shaowei Wang, swwang@lps.ecnu.edu.cn; Zhipei Sun, zhipei.sun@aalto.fi

Sci. Adv. **11**, eadz0106 (2025)
DOI: 10.1126/sciadv.adz0106

This PDF file includes:

Supplementary Text
Figs. S1 to S8
References

Supplementary Text

Section S1. The Universal Theory of GTPP Excitation.

TPP was first proposed by M. Kaliteevski (6). However, the TPP excitation condition $r_1 * r_2$=1 derived by them is based on semi-infinite photonic crystals and ideal negative-permittivity materials, and thus cannot be applied to determine the conditions for TPP excitation between finite photonic crystals and metal films. Therefore, we propose a condition for generating GTPP, $r_1 = r_2^*$, which will be demonstrated through the transfer matrix method (43) and Maxwell's equations.

Firstly, for any thin-film (multilayer) structure, it can be divided into two parts: the upper and lower parts, and can be equivalently regarded as an upper reflective interface (modulation interface) and a lower reflective interface (substrate-equivalent base interface), with a metal film existing at the interface, as shown in Fig.S1. Meanwhile, it has been proven that when the upper layer film stack is lossless, the total reflection coefficient r_{total} is only related to the properties of the interfaces (44).

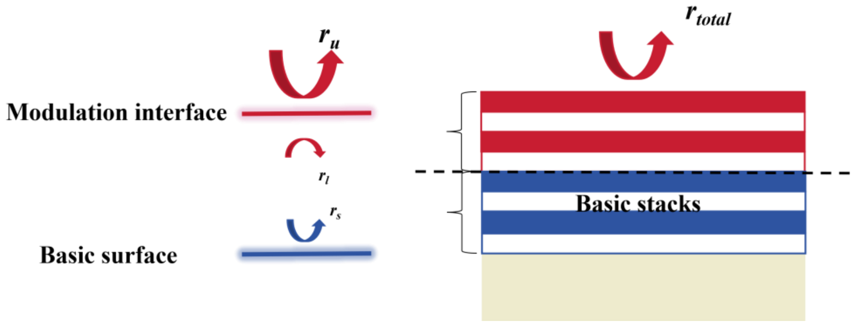

Fig.S1. GTPP model based on equivalent interface.

According to the transfer matrix method, for an arbitrary multilayer thin-film structure $m_1 m_2 \ldots m_i m_k$, the equivalent interface matrix can be written as:

$$\begin{bmatrix} \mu \\ v \end{bmatrix} = \left\{ \prod_{j=1}^{k} \begin{bmatrix} \cos\delta_j & \frac{i}{n_j}\sin\delta_j \\ i\eta_j \sin\delta_j & \cos\delta_j \end{bmatrix} \right\} \begin{bmatrix} 1 \\ \eta_0 \end{bmatrix} \quad \text{(S1)}$$

Here, δ_j represents the phase thickness of the j-th layer, where j=0, 1, 2, ….

$$\delta_j = \frac{2\pi}{\lambda}\eta_j L\cos\theta_j \quad \text{(S2)}$$

n_j is the admittance of the m_j-th layer, λ represents the wavelength of the incident light in vacuum, and θ_j is the angle of incidence in the m_j-th layer. For the sake of simplicity, Equation (S1) can be rewritten as:

$$\begin{bmatrix} \mu \\ v \end{bmatrix} = \left(\prod_{j=1}^{K} M_j \right) \begin{bmatrix} 1 \\ \eta_0 \end{bmatrix} \quad \text{(S3)}$$

At this time, the equivalent admittance of the equivalent interface Y can be expressed as:

$$Y = \frac{v}{\mu} \qquad \text{(S4)}$$

Similarly, Y can also be represented as

$$Y = \alpha + i\beta \qquad \text{(S5)}$$

Next, it is necessary to first prove a lemma to pave the way for the GTPP generation theory

Lemma: When light with a specific angle and wavelength is incident on the substrate surface, only

one set of multilayer films (or equivalent medium layers) can make the total reflectivity of a substrate zero. Here, substrates with the same optical admittance η are considered to be the same substrate.

The substrate-equivalent base interface can be mathematically expressed as $\alpha\begin{bmatrix}1\\ \eta\end{bmatrix}$, where $\alpha \neq 0$.

Assume there are two base interfaces, $\alpha\begin{bmatrix}1\\ \eta_1\end{bmatrix}$ and $\beta\begin{bmatrix}1\\ \eta_2\end{bmatrix}$, where $\eta_1 \neq \eta_2, \alpha \neq 0, \beta \neq 0$. For these two base interfaces, there is the same set of film layers, denoted as $\prod_{i=1}^{k} A_i$, which makes the total reflectivity of these two base interfaces zero.

$$\left(\prod_{i=1}^{k} A_i\right)\alpha\begin{bmatrix}1\\ \eta_1\end{bmatrix} = \begin{bmatrix}1\\ \eta_0\end{bmatrix} \qquad \text{(S6)}$$

$$\left(\prod_{i=1}^{k} A_i\right)\beta\begin{bmatrix}1\\ \eta_2\end{bmatrix} = \begin{bmatrix}1\\ \eta_0\end{bmatrix} \qquad \text{(S7)}$$

where η_1 and η_2 are the admittances of the two base interfaces, η_0 is the admittance of the incident medium, $\alpha \neq 0, \beta \neq 0$, respectively.

Subtracting Equation (S7) from Equation (S6), we obtain

$$\left(\prod_{i=1}^{k} A_i\right)\alpha\begin{bmatrix}\alpha-\beta\\ \alpha\eta_1-\beta\eta_2\end{bmatrix} = \begin{bmatrix}0\\ 0\end{bmatrix} \qquad \text{(S8)}$$

The right-hand side of the equation is 0. Noting that $\eta_1 \neq \eta_2, \alpha \neq 0, \beta \neq 0$, the rank of the matrix $\prod_{i=1}^{k} A_i$ must be 0. For Equation (S8) to hold, $\prod_{i=1}^{k} A_i$ must not be full rank. However, $\text{rank}(A_i) = 2$, so the above assumption does not hold.

Therefore, the lemma is proved.

Next, to prove $r_{total} = 0 \Leftrightarrow r_l = r_s^*$, it is necessary to prove the following two theorems:

Theorem 1: When the multilayer film is a non-absorptive medium, if $r_l = r_s^*$, then $r_{total} = 0$.

Theorem 2: When the multilayer film is a non-absorptive medium, if $r_{total} = 0$, then $r_l = r_s^*$.

The proof of Theorem 1 is as follows:

Assume there is a multilayer film $u_1u_2\cdots u_i\cdots u_k$, then its equivalent admittance Y as the modulating interface can be written as

$$C_1\begin{bmatrix}1\\ Y_1\end{bmatrix} = \left\{\prod_{j=k}^{1}\begin{bmatrix}\cos\delta_j & \frac{i}{\eta_j}\sin\delta_j\\ i\eta_j\sin\delta_j & \cos\delta_j\end{bmatrix}\right\}\begin{bmatrix}1\\ \eta_0\end{bmatrix} \qquad \text{(S9)}$$

where C_1 is a constant, η_j is the admittance of the u_j layer, η_0 is the admittance of the incident medium, and η_0 is a positive real number.

The reflection coefficient of the lower surface can be obtained as:

$$r_l = \frac{\eta_0 - Y_1}{\eta_0 + Y_1} \qquad \text{(S10)}$$

Since $Y_1 = Y_2^*$, it follows that $r_1 = r_s^*$.

The admittance of the substrate can be written as:

$$C_2\begin{bmatrix}1\\Y_2\end{bmatrix}=\left\{\left\{\prod_{j=k}^{1}\begin{bmatrix}\cos\delta_j & \frac{i}{\eta_j}\sin\delta_j\\ i\eta_j\sin\delta_j & \cos\delta_j\end{bmatrix}\right\}\begin{bmatrix}1\\\eta_0\end{bmatrix}\right\}^* \qquad (S11)$$

Since the incident medium and the multilayer film are non-absorptive, η_0 is a real number, therefore

$$C_2\begin{bmatrix}1\\Y_2\end{bmatrix}=\left\{\left\{\prod_{j=k}^{1}\begin{bmatrix}\cos\delta_j & \frac{i}{\eta_j}\sin\delta_j\\ i\eta_j\sin\delta_j & \cos\delta_j\end{bmatrix}\right\}\begin{bmatrix}1\\\eta_0\end{bmatrix}\right\}^*$$

$$=\left\{\prod_{j=k}^{1}\begin{bmatrix}\cos\delta_j & \frac{i}{\eta_j}\sin\delta_j\\ i\eta_j\sin\delta_j & \cos\delta_j\end{bmatrix}^*\right\}\begin{bmatrix}1\\\eta_0\end{bmatrix} \qquad (S12)$$

The transfer matrix of the entire thin-film structure can be expressed as:

$$\begin{bmatrix}\mu\\\nu\end{bmatrix}=\left\{\prod_{j=k}^{1}\begin{bmatrix}\cos\delta_j & \frac{i}{\eta_j}\sin\delta_j\\ i\eta_j\sin\delta_j & \cos\delta_j\end{bmatrix}\right\}\begin{bmatrix}1\\Y_2\end{bmatrix}$$

$$=\frac{1}{C_2}\left\{\prod_{j=k}^{1}\begin{bmatrix}\cos\delta_j & \frac{i}{\eta_j}\sin\delta_j\\ i\eta_j\sin\delta_j & \cos\delta_j\end{bmatrix}\right\}\left\{\prod_{j=k}^{1}\begin{bmatrix}\cos\delta_j & \frac{i}{\eta_j}\sin\delta_j\\ i\eta_j\sin\delta_j & \cos\delta_j\end{bmatrix}^*\right\}\begin{bmatrix}1\\\eta_0\end{bmatrix}=\frac{1}{C_2}\begin{bmatrix}1\\\eta_0\end{bmatrix} \ (S13)$$

The total equivalent admittance Y_{total} can be written as

$$Y_{\text{total}}=\frac{\nu}{\mu}=\eta_0 \qquad (S14)$$

Therefore, the total reflection coefficient $r_{\text{total}}=0$, and Theorem 1 is proved.

According to the lemma proved earlier, the substrate is unique, and the dielectric multilayer film (dielectric film stack) that makes the substrate's reflectivity zero is also unique. Therefore, Theorem 2 is also proved.

From the above proof, we find that for any substrate (non-absorptive medium, metal, and its constructed film stack) with an equivalent base interface, as long as the corresponding top film stack and incident medium are non-absorptive, the conclusion $r_l=r_s^*\Leftrightarrow r_{\text{total}}=0$ holds, and GTPP can be excited at the metal interface.

Section S2. The influence of different ultra-thin metals on GTPP excitation.

In this section, we will investigate the impact of different metal types. The film stack structure is Sub|$(HL)^{11}ML(HL)^3$, where H represents a 62 nm thick Ta_2O_5 layer, L denotes a 91 nm thick SiO_2 layer, and M corresponds to a 2 nm thick Au, Ag and Al film. As shown in Figure S4, the purple, dark green, and cyan lines represent the reflectance spectra of the GTPP devices with Au (45), Ag (46), and Al (47), respectively. The black, red, and bright green lines represent the transmittance spectra of the above three metals, respectively. It can be seen from the figure that the structure containing the Au layer has the highest absorption rate, and the peak position is closest to the experimentally measured absorption peak at 532 nm. For the structure containing a thick Ag layer, the TPP mode excited is generally consistent with that of Au (48), but for the ultrathin metal, the peak position of the structure containing the Ag layer is red-shifted, and the absorption peak is reduced. The possible reason is that their optical properties are quite different at this time, leading to changes in the excited GTPP mode, the higher refractive index of Ag leads to an increase in the

equivalent optical path length ($nLcos\theta$). For the Al layer, its higher metal loss results in a broader GTPP mode, and the difference in metal properties leads to lower absorption.

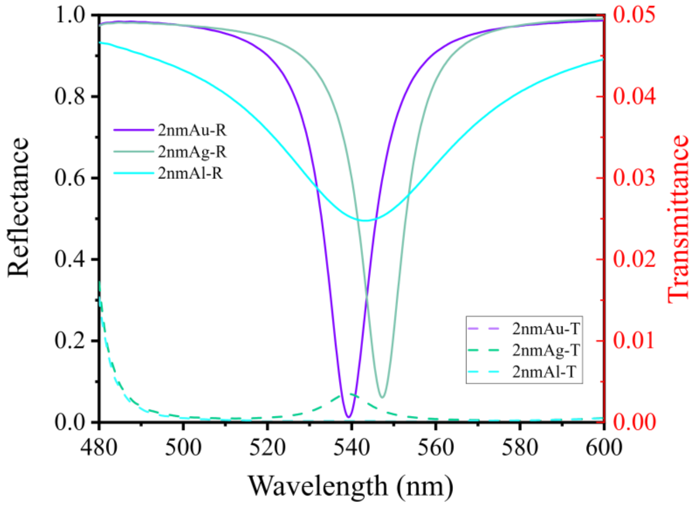

Fig.S2. The reflection and transmission spectrum with different metals.

Section S3. Spectra of GTPP devices as a function of the incident angle

For TPP, an important characteristic is that its dispersion curve exhibits a parabolic linearity. Typically, the dispersion curve of TPP is obtained through angle-resolved spectroscopy with respect to the incident light angle. For the structure Sub|$(HL)^{11}ML(HL)^{3}$, the angle-resolved spectroscopy of the excited GTPP is shown in Figure S5, where (a) displays the calculated reflectance of TM waves (p-polarized light) as a function of the incident angle, and (b) corresponds to the reflectance of TE waves (s-polarized light) as a function of the angle. It can be seen that as the incident angle increases, the equivalent optical path length of the DBR gradually shortens. At this time, the central peak position excited by GTPP will undergo a blue shift with the increase of the incident angle, and it can be seen that TM and TE waves have the same variation law. It is worth noting that for TM waves, as the incident angle increases, the equivalent refractive index of the DBR also increases, resulting in a narrower full width at half maximum (FWHM) of GTPP. For TE waves, however, as the incident angle increases, the equivalent refractive index of the DBR decreases, and therefore its FWHM will broaden.

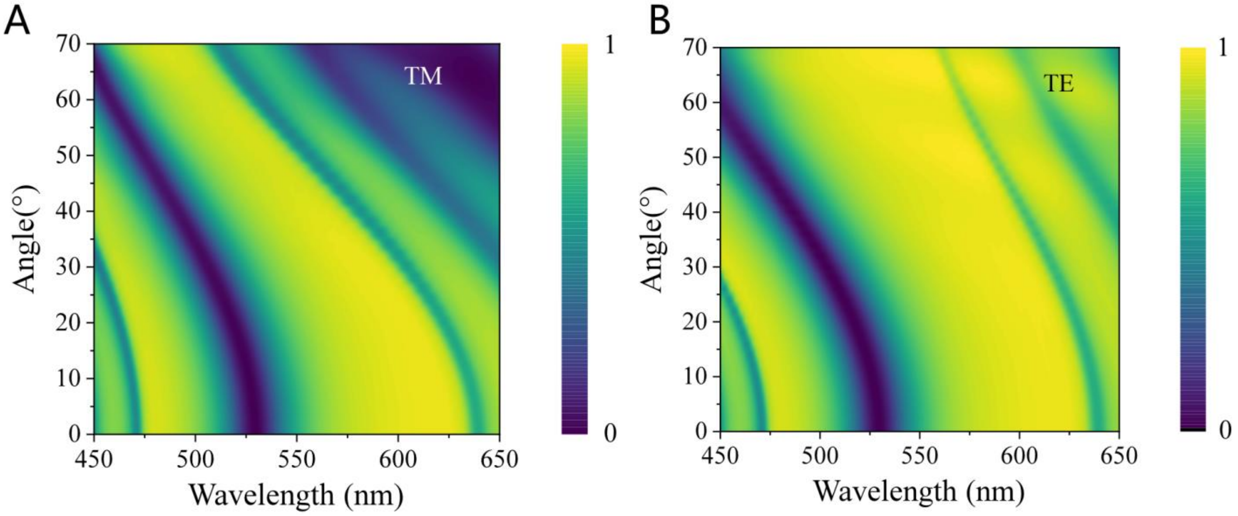

Fig.S3. Influence of incident Angle and polarization on reflection peak (a) The reflectance of TM wave varies with incident Angle; (b) The reflectance of TE wave varies with the incidence Angle.

Section S4. The electromagnetic field distribution and reflection phase of the GTPP device.

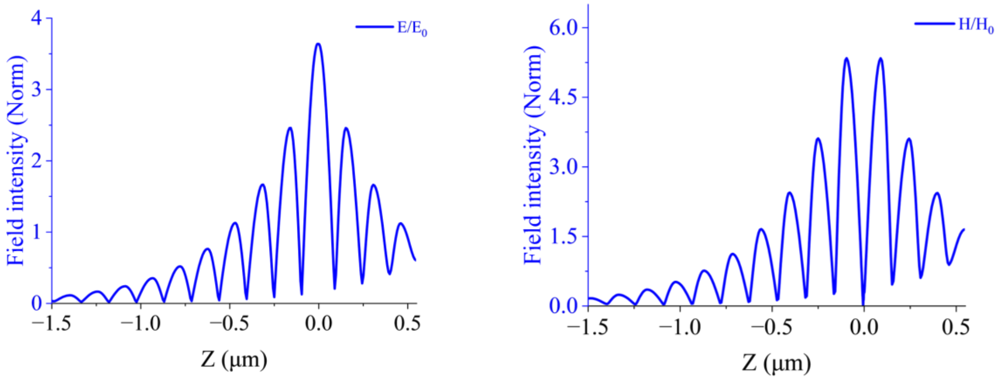

Fig.S4. The distribution of electric and magnetic field strengths of the GTPP device at the resonance peak (532 nm). The multilayer structure Sub|$(HL)^{11}ML(HL)^{3}$, where H represents a 62 nm thick Ta_2O_5 layer, L denotes a 91 nm thick SiO_2 layer, and M corresponds to a 2 nm thick Au film, M is located at Z = 0.

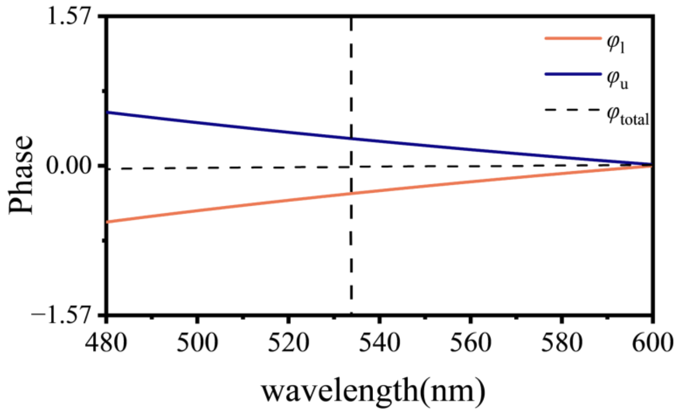

Fig.S5. The phase variation of the GTPP device with wavelength. φ_l represents the reflection phase of the equivalent lower interface, φ_u is the reflection phase of the equivalent upper interface, and φ_{total}=φ_l+φ_u is the total reflection phase. The dashed line in the figure indicates where φ_{total} =0, representing that φ_l and φ_u are opposite numbers at this time.

Section S5. Phase diagrams and absorption spectra of different metal thicknesses

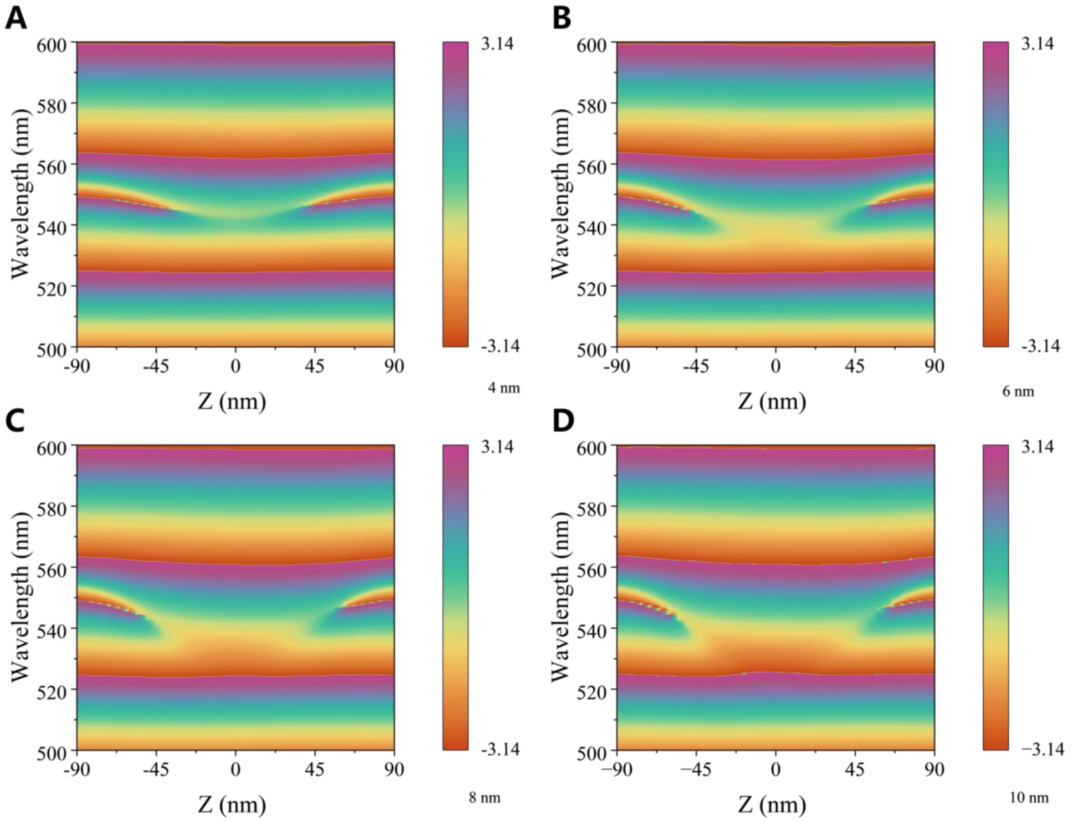

Fig. S6. Evolution of phase singularities under different metal thicknesses. (A) 4 nm Au film. (B) 6 nm Au film. (C) 8 nm Au film. (D) 10 nm Au film.

Section S6. Schematic diagram of the measurement setup.

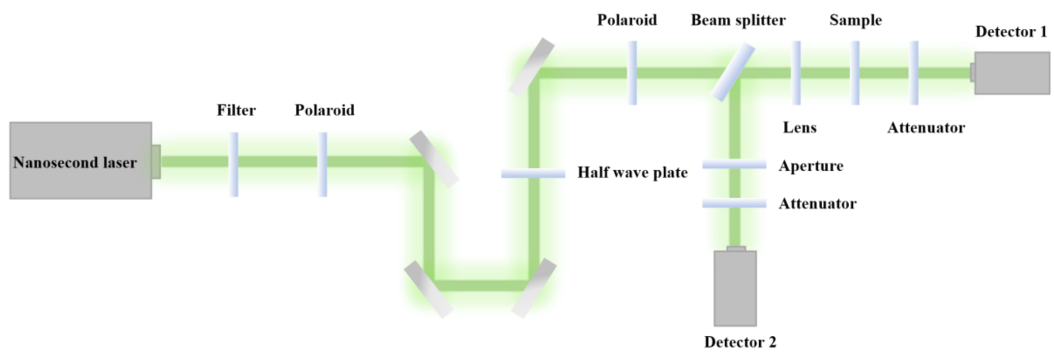

Fig.S7. Optical path of reflective power scanning test.

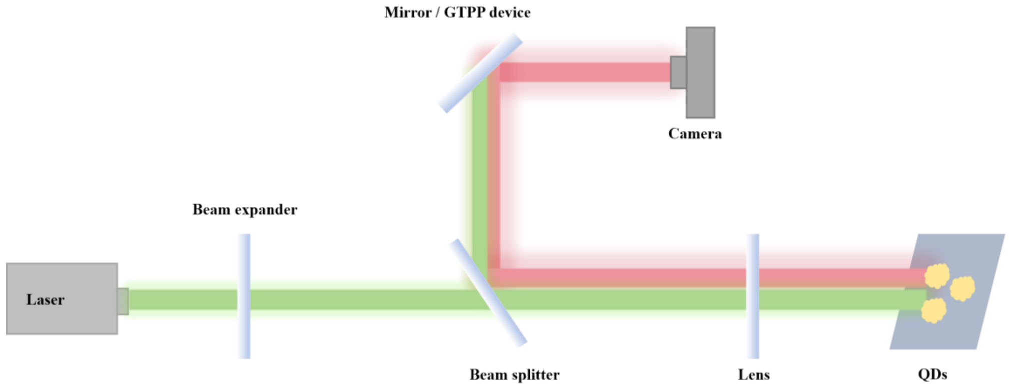

Fig.S8. Schematic diagram of microfluorescence detection system.

Section S7. Detailed Fabrication Process of the perfect absorber and GTPP

The fabrication process of the perfect absorber based on ultrathin metal-dielectric interface modulation are as following steps: (1) The K9 glass substrate was ultrasonically cleaned in acetone for 5 min, then transferred to isopropanol for an additional 5 min of ultrasonic cleaning. Subsequently, the substrate was thoroughly rinsed with deionized water to remove residual traces of isopropanol. Finally, the substrate was removed and dried with a nitrogen gun. (2) The cleaned K9 substrate was loaded into a high-vacuum electron beam evaporation system (Leybold ARES 1110), evacuated to 2×10^{-5} mbar, and heated to 230°C with a 600-second temperature stabilization period. Using a quartz crystal monitor (6 MHz crystal frequency), a 62 nm Ta_2O_5 layer was deposited at 0.2 nm/s followed by a 91 nm SiO_2 layer at 0.6 nm/s to complete the bottom distributed Bragg reflector (DBR) fabrication. (3) The substrate with the bottom DBR was loaded into a magnetron sputtering deposition system (K. J. Lesker ProLine PVD75) for deposition of a 2 nm metallic Au layer, with process parameters including: base pressure of 6.8×10^{-7} Torr, deposition power of 100 W, Ar pressure of 3 mTorr, and deposition rate of 0.29 nm/s. (4) Finally, the Au-coated sample was loaded into the high-vacuum electron beam evaporation system, evacuated to 2×10^{-5} mbar, and heated to 45°C. A 91 nm SiO_2 layer was deposited at 0.6 nm/s followed by a 62 nm Ta_2O_5 layer at 0.2 nm/s, yielding the Tamm plasmon polariton (TPPs) device with the (HL)11 ML(HL)3 structure.

The fabrication process of the GTPP device with $(HL)^{11}ML(HL)^{3}$ multilayer structure mainly includes the following steps: (1) The K9 glass substrate was first ultrasonically cleaned in acetone for 5 minutes, then transferred to isopropanol for another 5 minutes of ultrasonic cleaning. Subsequently, the substrate was thoroughly rinsed with deionized water to remove any residual isopropanol. Finally, the substrate was taken out and dried using a nitrogen gun. (2) The cleaned

K9 substrate was placed into a high-vacuum electron beam evaporation system (Leybold ARES 1110). The chamber was evacuated to 2×10^{-5} mbar and heated to 230°C, maintaining this temperature for 600 s. Using a quartz crystal monitor system (6 MHz crystal frequency), the Ta_2O_5 and SiO_2 layers were sequentially deposited at rates of 0.2 nm/s and 0.6 nm/s, respectively, with thicknesses of 62 nm for Ta_2O_5 and 91 nm for SiO_2, thus completing the bottom DBR fabrication. (3) The substrate with bottom DBR was then transferred to a magnetron sputtering deposition system (K. J. Lesker ProlinePVD75) for the deposition of a 2 nm Au metal layer. The process parameters included: base pressure of 6.8×10^{-7} Torr, deposition power of 100 W, Ar pressure of 3 mTorr, and deposition rate of 0.29 nm/s. (4) Finally, the Au-coated sample was placed back into the high-vacuum electron beam evaporation system. The chamber was evacuated to 2×10^{-5} mbar and heated to 45 °C. The 91 nm SiO_2 layer and 62 nm Ta_2O_5 layer were sequentially deposited at rates of 0.6 nm/s and 0.2 nm/s respectively. This completed the fabrication of the GTPP device with $(HL)^{11}ML(HL)^3$ structure.

First, the basic structure of the bottom DBR + Au film + phase modulation layer is prepared, denoted as $(HL)^{11}M2L$ (where 2L represents a 180nm-thick SiO_2 phase modulation layer). Through four cycles of ultraviolet lithography (using ML3 Aligner system) and inductively coupled plasma etching (using Oxford PlasmaPro System 100), 14 stepped cavity layers with different thicknesses can be fabricated, thereby forming a complete 14-channel integrated GTPP filter array, with each channel measuring 250μm×250μm. In detail, the fabrication of the 14-channel TPPS device utilizes sequential etching technology. Following the deposition of the 180-nm-thick phase modulation layer, each lithography-etching cycle constitutes a complete processing sequence. In this process, the lithography step creates localized masks through photoresist patterning, while the etching step selectively thins the film thickness in unmasked regions by material removal. Executing the first processing cycle produces a 2-channel device, the second cycle yields a 4-channel device, and following this progression, N cycles theoretically generate stepped cavity layers with 2^N distinct channels. Thus, four cycles of UV lithography and inductively coupled plasma (ICP) etching create 16 stepped cavity thicknesses. However, due to etching non-uniformity causing unintentional spectral overlap between two channel groups, the final implementation resulted in a 14-channel TPPS device. The specific experimental procedure requires the following modifications to step (4) in the aforementioned fabrication process: (4) Place the gold-coated sample into the high-vacuum electron beam evaporation equipment, evacuate to 2×10^{-5} mbar, heat the chamber to 45 °C, and deposit a 180 nm-thick SiO_2 layer as the phase modulation layer. (5) Fabricate the device array using a laser direct writing system: Spin-coat AZ5214 photoresist on the SiO_2-Au-DBR sample surface with spin parameters of 500 rpm for 5 seconds followed by 3000 rpm for 30 seconds, resulting in a photoresist thickness of approximately 2 μm; then perform pre-baking at 100 °C for 270 seconds; conduct the first UV exposure using Durham Magneto Optics Ltd's Micro-writer ML3 laser direct writing system to obtain dual-channel array patterns; develop the exposed sample in NMD-3 developer for 35 seconds, rinse with deionized water for 1 minute, and dry the sample surface with photoresist patterns using nitrogen gun. (6) Place the sample with photoresist patterns into the inductively coupled plasma etching system (Oxford's Plasmalab System 100), and perform the first SiO2 layer etching at 5.6×10^{-9} Torr. After resist removal, repeat steps (5)-(6) three more times according to the designed mask pattern to ultimately obtain 14 stepped SiO_2 layer structures. (7) Finally, place the etched sample into the high-vacuum electron beam evaporation equipment and sequentially deposit a 62 nm-thick Ta_2O_5 layer (at 0.2 nm/s) and a 91 nm-thick SiO_2 layer (at 0.6 nm/s), totaling 3 DBR pairs, to ultimately obtain the 14-channel integrated GTPP device.

Section S8. The key parameters of the experimental equipment.

First, the ARES 1110 instrument was manufactured in strict compliance with international standards, including the EU Machinery Directive (98/37/EG) and relevant EN/IEC specifications (EN 60204-1, DIN/EN 12100). The system integrates multiple components (vacuum chamber, pumping system, etc.) with a total weight of 3380 kg, whose technical parameters are documented in the manufacturer's specifications and installation drawings. For optimal operation, the system requires three-phase power supply (400V, 50Hz), process gases (oxygen, argon, etc.), compressed air (1.0-1.5 bar), and cooling water with controlled purity (pH 7.5-8.5, conductivity $\leq$600 μS/cm) to prevent scaling and corrosion. The vacuum system achieves an ultimate pressure of 1×10^{-6} mbar, with pumping speeds up to 1500 m^3/h (turbomolecular pump). Environmental conditions during operation are maintained at 15-30°C, 30-70% relative humidity, and minimal vibration (<0.1 mm/s). Exhaust emissions are treated to comply with environmental regulations, and operational noise remains below 75 dB(A) at 1-meter distance.

Second, the specification details of laser direct writing system (Durham Magneto Optics Ltd, Micro-writer ML3). The system features direct-writing resolutions of 0.6 μm, 1 μm, 2 μm and 5 μm, with corresponding writing speeds of 25 mm^2/min at 0.6 μm resolution, 50 mm^2/min at 1 μm resolution, 100 mm^2/min at 2 μm resolution, and 180 mm^2/min at 5 μm resolution, while achieving multilayer overlay alignment accuracy of 0.5 μm, optical profilometer z-axis resolution of 100 nm, fine grid accuracy of 100 nm, and a minimum stage step size of 50 nm.

Finally, the specification details of Cary 7000 UV-Vis-NIR spectrometer. The spectrometer features a measurement range of 190-3300 nm, equipped with PMT and PbS detectors, and supports transmission, absorption, scattering, and absolute reflectance measurement modes.

REFERENCES AND NOTES

1. H. Ohno, E. E. Mendez, A. Alexandrou, J. M. Hong, Tamm states in superlattices. *Surf. Sci.* **267**, 161–165 (1992).

2. A. M. Merzlikin, M. Inoue, A. P. Vinogradov, A. V. Dorofeenko, A. B. Granovsky, A. A. Lisyansky, Tamm state at one-dimensional photonic crystals. *J. Magn. Soc. Jpn.* **30**, 616–619 (2006).

3. J.-Y. Guo, Y. Sun, H.-Q. Li, Y.-W. Zhang, H. Chen, Optical Tamm states in dielectric photonic crystal heterostructure. *Chin. Phys. Lett.* **25**, 2093–2096 (2008).

4. T. Goto, A. V. Dorofeenko, A. M. Merzlikin, A. V. Baryshev, A. P. Vinogradov, M. Inoue, A. A. Lisyansky, A. B. Granovsky, Optical tamm states in one-dimensional magnetophotonic structures. *Phys. Rev. Lett.* **101**, 113902 (2008).

5. J. Guo, Y. Sun, Y. Zhang, H. Li, H. Jiang, H. Chen, Experimental investigation of interface states in photonic crystal heterostructures. *Phys. Rev. E* **78**, 026607 (2008).

6. M. Kaliteevski, I. Iorsh, S. Brand, R. A. Abram, J. M. Chamberlain, A. V. Kavokin, I. A. Shelykh, Tamm plasmon-polaritons: Possible electromagnetic states at the interface of a metal and a dielectric Bragg mirror. *Phys. Rev. B* **76**, 165415 (2007).

7. M. E. Sasin, R. P. Seisyan, M. A. Kalitteevski, S. Brand, R. A. Abram, J. M. Chamberlain, A. Y. Egorov, A. P. Vasil'ev, V. S. Mikhrin, A. V. Kavokin, Tamm plasmon polaritons: Slow and spatially compact light. *Appl. Phys. Lett.* **92**, 251112 (2008).

8. S. H. Tsang, S. F. Yu, X. F. Li, H. Y. Yang, H. K. Liang, Observation of Tamm plasmon polaritons in visible regime from ZnO/Al_2O_3 distributed Bragg reflector - Ag interface. *Opt. Commun.* **284**, 1890–1892 (2011).

9. Y.-t. Fang, L.-k. Chen, N. Zhu, J. Zhou, Tamm states of one-dimensional metal-dielectric photonic crystal. *IET Optoelectron.* **7**, 9–13 (2013).

10. Y. Chen, D. Zhang, L. Zhu, Q. Fu, R. Wang, P. Wang, H. Ming, R. Badugu, J. R. Lakowicz, Effect of metal film thickness on Tamm plasmon-coupled emission. *Phys. Chem. Chem. Phys.* **16**, 25523–25530 (2014).

11. H. Zhou, G. Yang, K. Wang, H. Long, P. Lu, Multiple optical Tamm states at a metal-dielectric mirror interface. *Opt. Lett.* **35**, 4112–4114 (2010).

12. C.-Y. Chang, Y.-H. Chen, Y.-L. Tsai, H.-C. Kuo, K.-P. Chen, Tunability and optimization of coupling efficiency in tamm plasmon modes. *IEEE J. Sel. Top. Quantun Electron.* **21**, 262–267 (2015).

13. L. Li, H. Zhao, J. Zhang, Electrically tuning reflection of graphene-based Tamm plasmon polariton structures at 1550 nm. *Appl. Phys. Lett.* **111**, 083504 (2017).

14. P. S. Pankin, V. S. Sutormin, V. A. Gunyakov, F. V. Zelenov, I. A. Tambasov, A. N. Masyugin, M. N. Volochaev, F. A. Baron, K. P. Chen, V. Y. Zyryanov, S. Y. Vetrov, I. V. Timofeev, Experimental implementation of tunable hybrid Tamm-microcavity modes. *Appl. Phys. Lett.* **119**, 161107 (2021).

15. Y.-t. Fang, Y.-x. Ni, H.-q. He, J.-x. Hu, Effect of hybrid state of surface plasmon-polaritons, magnetic defect mode and optical Tamm state on nonreciprocal propagation. *Opt. Commun.* **320**, 99–104 (2014).

16. G. Lu, K. Zhang, Y. Zhao, L. Zhang, Z. Shang, H. Zhou, C. Diao, X. Zhou, Perfect optical absorbers by all-dielectric photonic crystal/metal heterostructures due to optical Tamm state. *Nanomaterials* **11**, 3447 (2021).

17. Y. Gong, X. Liu, H. Lu, L. Wang, G. Wang, Perfect absorber supported by optical Tamm states in plasmonic waveguide. *Opt. Express* **19**, 18393–18398 (2011).

18. R. Das, T. Srivastava, R. Jha, Tamm-plasmon and surface-plasmon hybrid-mode based refractometry in photonic bandgap structures. *Opt. Lett.* **39**, 896–899 (2014).

19. W. L. Zhang, F. Wang, Y. J. Rao, Y. Jiang, Novel sensing concept based on optical Tamm plasmon. *Opt. Express* **22**, 14524–14529 (2014).

20. C. Zhang, K. Wu, V. Giannini, X. Li, Planar hot-electron photodetection with Tamm plasmons. *ACS Nano* **11**, 1719–1727 (2017).

21. W. Liang, Z. Xiao, H. Xu, H. Deng, H. Li, W. Chen, Z. Liu, Y. Long, Ultranarrow-bandwidth planar hot electron photodetector based on coupled dual Tamm plasmons. *Opt. Express* **28**, 31330–31344 (2020).

22. C. Symonds, G. Lheureux, J. P. Hugonin, J. J. Greffet, J. Laverdant, G. Brucoli, A. Lemaitre, P. Senellart, J. Bellessa, Confined Tamm plasmon lasers. *Nano Lett.* **13**, 3179–3184 (2013).

23. G. Lheureux, S. Azzini, C. Symonds, P. Senellart, A. Lemaître, C. Sauvan, J.-P. Hugonin, J.-J. Greffet, J. Bellessa, Polarization-controlled confined Tamm plasmon lasers. *ACS Photonics* **2**, 842–848 (2015).

24. P. Zhao, W. T. Su, R. Wang, X. F. Xu, F. S. Zhang, Properties of thin silver films with different thickness. *Phys. E* **41**, 387–390 (2009).

25. H. Liu, B. Wang, E. S. P. Leong, P. Yang, Y. Zong, G. Si, J. Teng, S. A. Maier, Enhanced surface plasmon resonance on a smooth silver film with a seed growth layer. *ACS Nano* **4**, 3139–3146 (2010).

26. M. Mayy, G. Zhu, E. Mayy, A. Webb, M. A. Noginov, Low temperature studies of surface plasmon polaritons in silver films. *J. Appl. Phys.* **111**, 094103 (2012).

27. L. Ke, S. C. Lai, H. Liu, C. K. N. Peh, B. Wang, J. H. Teng, Ultrasmooth silver thin film on PEDOT:PSS nucleation layer for extended surface plasmon propagation. *ACS Appl. Mater. Interfaces* **4**, 1247–1253 (2012).

28. Y. Zhang, Z. Li, F. Cao, T. Tang, T. Ding, Ultra-wide stopband polarization narrow band filter combining optical Tamm state engineering and grating structure. *J. Nanophotonics* **18**, 046006 (2024).

29. J. Zhang, L. Long, Y. Wu, H. Ye, M. Liu, Metafilms for visible and infrared compatible camouflage of high-temperature targets. *Mater Res Express* **10**, 036402 (2023).

30. C. C. Chang, T. Y. Chen, T. W. Lin, J. F. Leng, K. Tamada, Y. J. Lee, Flexible and ultranarrow transmissive color filters by simultaneous excitations of triple resonant eigenmodes in hybrid metallic-optical tamm state devices. *ACS Photonics* **8**, 540–549 (2021).

31. A. Ciesielski, L. Skowronski, M. Trzcinski, T. Szoplik, Controlling the optical parameters of self-assembled silver films with wetting layers and annealing. *Appl. Surf. Sci.* **421**, 349–356 (2017).

32. C. Kar, S. Jena, D. V. Udupa, K. D. Rao, Tamm plasmon polariton in planar structures: A brief overview and applications. *Opt. Laser Technol.* **159**, 108928 (2023).

33. K. V. Sreekanth, J. Perumal, U. S. Dinish, P. Prabhathan, Y. Liu, R. Singh, M. Olivo, J. Teng, Tunable Tamm plasmon cavity as a scalable biosensing platform for surface enhanced resonance Raman spectroscopy. *Nat. Commun.* **14**, 7085 (2023).

34. S. Y. Vetrov, P. S. Pankin, I. V. Timofeev, The optical Tamm states at the interface between a photonic crystal and a nanocomposite containing core-shell particles. *J. Optics* **18**, 065106 (2016).

35. C. Zhang, Q.-Y. Huang, Q. Cui, C. Ji, Z. Zhang, X. Chen, T. George, S. Zhao, L. J. Guo, High-performance large-scale flexible optoelectronics using ultrathin silver films with tunable properties. *ACS Appl. Mater. Interfaces* **11**, 27216–27225 (2019).

36. K. M. Tsysar, E. M. Smelova, A. M. Saletsky, V. G. Andreev, Quantum size effect in conductive properties of silver nanofilms. *Thin Solid Films* **710**, 138263 (2020).

37. K. Xu, J. Zhang, B. Yang, F. Wu, C. Yin, Non-reciprocal optical Tamm state in a photonic crystal heterojunction containing Weyl semimetals. *Phys. B. Condens. Matter* **691**, 416329 (2024).

38. B. Abeles, J. I. Gittleman, Composite material films: Optical properties and applications. *Appl. Optics* **15**, 2328–2332 (1976).

39. X. Guan, Q. Liu, C. Li, Z. Yin, J. Wu, P. Yu, W. Lu, S. Wang, Generalized Fano resonance theory based on Fabry-Perot cavity. *J. Phys. D. Appl. Phys.* **57**, 135102 (2024).

40. Z. Xuan, J. Li, Q. Liu, F. Yi, S. Wang, W. Lu, Artificial structural colors and applications. *The Innovation* **2**, 100081 (2021).

41. L. Leandro, R. Malureanu, N. Rozlosnik, L. Lavrinenko, Ultrathin, ultrasmooth gold layer on dielectrics without the use of additional metallic adhesion layers. *ACS Appl. Mater. Interfaces* **7**, 5797–5802 (2015).

42. M. Liu, W. Chen, G. Hu, S. Fan, D. N. Christodoulides, C. Zhao, C.-W. Qiu, Spectral phase singularity and topological behavior in perfect absorption. *Phys. Rev. B* **107**, L241403 (2023).

43. D. Vincent, Optical limiting threshold in carbon suspensions and reverse saturable absorber materials. *Appl. Optics* **40**, 6646–6653 (2001).

44. H. A. Macleod, *Thin-Film Optical Filters* (CRC Press, ed. 3, 2001).

45. Q. Liu, X. Zhao, C. Li, X. Zhou, Y. Chen, S. Wang, W. Lu, Coupled Tamm plasmon polaritons induced narrow bandpass filter with ultra-wide stopband. *Nano Res* **15**, 4563–4568 (2022).

46. D. I. Yakubovsky, Y. V. Stebunov, R. V. Kirtaev, G. A. Ermolaev, M. S. Mironov, S. M. Novikov, A. V. Arsenin, V. S. Volkov, Ultrathin and ultrasmooth gold films on monolayer MoS_2. *Adv. Mater. Interfaces* **6**, 1900196 (2019).

47. J. Gong, R. Dai, Z. Wang, Z. Zhang, Thickness dispersion of surface plasmon of ag nano-thin films: Determination by ellipsometry iterated with transmittance method. *Sci. Rep.* **5**, 9279 (2015).

48. S. Gao, J. Lian, P. Song, P. Li, Z. Ma, X. Wang, S. Wu, “Study on optical constant of ultrathin aluminum films deposited by molecular beam epitaxy,” in *2011 Symposium on Photonics and Optoelectronics* (IEEE, 2011), pp. 4–4.
